# pH Induced Conformational Transitions in the Transforming Growth Factor β-Induced Protein (TGFβIp) Associated Corneal Dystrophy Mutants

**DOI:** 10.1038/srep23836

**Published:** 2016-03-31

**Authors:** Elavazhagan Murugan, Anandalakshmi Venkatraman, Zhou Lei, Victoria Mouvet, Rayne Rui Yi Lim, Nandhakumar Muruganantham, Eunice Goh, Gary Swee Lim Peh, Roger W. Beuerman, Shyam S. Chaurasia, Lakshminarayanan Rajamani, Jodhbir S. Mehta

**Affiliations:** 1 Tissue Engineering and Stem Cell Group, Singapore Eye Research Institute, Singapore; 2 Duke-NUS Graduate Medical School, Singapore; 3 Proteomics and Microanalysis laboratory, Singapore Eye Research Institute, Singapore; 4 Ocular Chemistry and Anti-Infectives, Singapore Eye Research Institute, Singapore; 5 Department of Ophthalmology, Yong Loo Lin School of Medicine, NUS, Singapore; 6 Singapore National Eye Centre, Singapore.

## Abstract

Most stromal corneal dystrophies are associated with aggregation and deposition of the mutated transforming growth factor-β induced protein (TGFβIp). The 4^th^_FAS1 domain of TGFβIp harbors ~80% of the mutations that forms amyloidogenic and non-amyloidogenic aggregates. To understand the mechanism of aggregation and the differences between the amyloidogenic and non-amyloidogenic phenotypes, we expressed the 4^th^_FAS1 domains of TGFβIp carrying the mutations R555W (non-amyloidogenic) and H572R (amyloidogenic) along with the wild-type (WT). R555W was more susceptible to acidic pH compared to H572R and displayed varying chemical stabilities with decreasing pH. Thermal denaturation studies at acidic pH showed that while WT did not undergo any conformational transition, the mutants exhibited a clear pH-dependent irreversible conversion from αβ conformation to β-sheet oligomers. The β-oligomers of both mutants were stable at physiological temperature and pH. Electron microscopy and dynamic light scattering studies showed that β-oligomers of H572R were larger compared to R555W. The β-oligomers of both mutants were cytotoxic to primary human corneal stromal fibroblast (pHCSF) cells. The β-oligomers of both mutants exhibit variations in their morphologies, sizes, thermal and chemical stabilities, aggregation patterns and cytotoxicities.

Corneal Dystrophies are inherited protein aggregation disorders characterized by the deposition of misfolded proteins aggregates in various layers of the cornea^1^,^2^,^3^. Most dystrophies in the corneal stromal region are associated with the mutations in the transforming growth factor β-induced protein (TGFβIp). TGFβIp aggregation and deposition occurs only in the cornea, though it is present in abundance in various connective tissues^4^,^5^,^6^,^7^. The mature TGFβIp, a 660aa protein has an N-terminal cysteine-rich EMILIN-like (EMI) domain, four fascicilin-like (FAS1) domains and an integrin-binding RGD motif at the C-terminus^8^. TGFβIp-associated corneal dystrophies are phenotypically heterogeneous, inherited in an autosomal dominant manner^1^,^9^,^10^ and are classified as lattice, granular, combined lattice and granular, Reis-Buckler and Thiel-Behnke corneal dystrophies^1^,^2^,^11^,^12^. So far, 64 single amino acid mutations associated with distinct phenotypes have been reported^4^,^13^,^14^. Among the four FAS1 domains of TGFβIp, the 1^st^ and 4th FAS1 domains carry the disease related mutations, with ~80% of the mutations residing in the 4^th^_FAS1 domain^11^. In lattice corneal dystrophies (LCD), the protein aggregates appear as lattice lines or amyloid fibrils (amyloidogenic). In granular corneal dystrophies (GCD), they appear as granular, non-amyloidogenic deposits^11^,^12^,^13^,^14^,^15^,^16^. Both phenotypes display significant differences in morphology, aggregation and tinctorial properties. However, the mechanisms adopted by these phenotypes in forming highly distinct ultrastructures, remains to be elucidated.

It has been reported that the ability to form highly ordered aggregates such as amyloids, resides within the polypeptide chains rather than the whole protein^17^. Hence, we chose to explore the crucial 4^th^_FAS1 domain (135aa) as a representative of the full-length TGFβIp. Bioinformatics analyses of the aggregation propensities of various regions of TGFβIp have also shown that the 4^th^_FAS1 domain harbors regions of high aggregation propensities^18^,^19^. Also, homology-based modelling studies have shown that the 4^th^_FAS1 domain displays the properties of the full-length TGFβIp^20^.

In our study, we aim to delineate the differences between the amyloidogenic and the non-amyloidogenic mutants and between the mutants and wild-type (WT) TGFβIp to explain the physiological variations exhibited by these phenotypes. We had previously reported the cloning, expression and purification of the 4^th^_FAS1 domain of four TGFβIp mutants^21^. Our studies indicated that under physiological pH, the mutants are more stable than the WT. Here, we chose to examine the effects of factors like temperature and pH on the properties of the 4^th^_FAS1 domains of TGFβIp harboring mutations of the amyloidogenic and non-amyloidogenic phenotypes. The amyloidogenic H572R (LCDI/IIIA), discovered in Thai^22^ and Chilean populations^23^ with ages of onset ranging around mid-twenties, is characterized by central sub-epithelial needle-like lattice lines and polymorphic anterior stromal opacities. The highly ubiquitous non-amyloidogenic R555W (GCDI/II)^24^,^25^,^26^ appears as rod shaped or granular bodies with sharp borders found in the central corneal stroma^27^,^28^. We aimed to examine the effects of these charge modifying mutations on the domains, their aggregation, their sensitivity to pH and temperature.

Aggregating proteins are found to be more susceptible to acidic pH and increase in temperature^29^,^30^. Even in TGFβIp, it has been shown that the R124H mutation induces localization of TGFβIp to lysosomes with an acidic environment^31^. In the present study, we have investigated the effects of acidic pH, denaturants and temperature on the secondary structure and conformational stability of the domains. The cytotoxicities of the aggregates were also studied in primary human corneal stromal fibroblasts (pHCSF).

## Results

### Effects of biochemical and biophysical factors on WT and mutants

**Figure 1:**
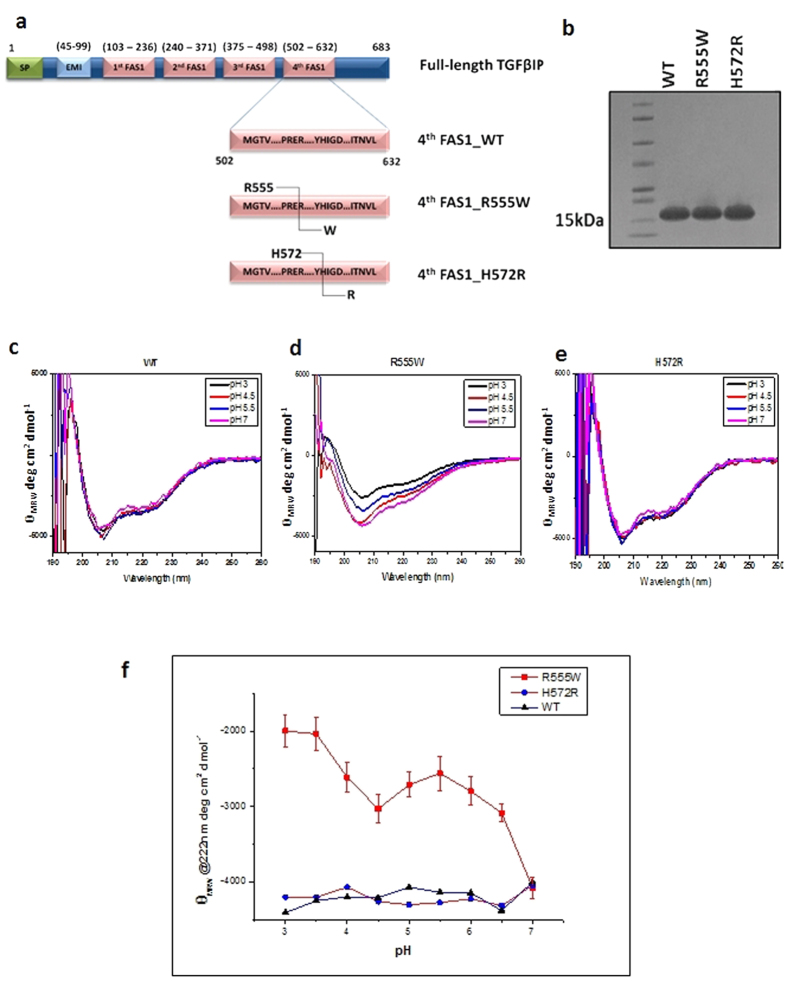
Biochemical and biophysical properties of the native 4th_FAS1 domains of the wild-type and mutant TGFβIp. (**a**) Schematic representation of the domain arrangement and boundaries of the full-length TGFβIp and 4^th^_FAS1 domains of the wild-type, non-amyloidogenic (R555W) and amyloidogenic (H572R) mutants used in the study. (**b**) SDS-PAGE gel showing the purified fractions of the 4^th^_FAS1 domains of the WT, R555W and H572R mutants. (**c–e**) Far UV CD spectra of the 4^th^_FAS1 domains of WT (**d**), R555W (**e**) and H572R (**f**) incubated for 16 hours at acidic pH conditions (pH 3, 4.5, 5.5 and 7). The R555W mutant displayed clear changes in the CD spectra at 222 nm and 207 nm with decrease in pH (**f**). The CD intensity at 222 nm decreased with decrease in pH confirming the unfolding of the secondary structures. The CD spectra for the WT (**c**) and H572R (**e**) mutant remained almost unchanged. Plotting the intensities at 222 nm at varying pH (**g**) showed that the non-amyloidogenic phenotype, R555W, was more sensitive to pH and the amyloidogenic phenotype, H572R, remained more stable to pH changes at room temperature.

The native 4^th^_FAS1 domain (Fig. 1a) of TGFβIp (Genbank_ID-NM_000358; Protein_ID:-NP_000349) and the mutants R555W and H572R were cloned and purified (Fig. 1b) as described previously^21^. The estimated pI values of the WT, R555W and H572R domains were 6.53, 6.32 and 6.65 respectively. The amino acid substitutions (R→W and H→R) were associated with changes in charge and hydrophobicity as listed in the table (Table 1).

### Effect of pH on the secondary structures of the WT and mutants

The CD spectra for the WT and the mutants at pH 7 (Fig. 1c–e) showed negative minima in the n – π* region (222 nm) and a weak shoulder at the π–π* region (207 nm) corresponding to their mixed α-helical and β-sheet conformations. Under acidic conditions, there were discernible differences between the WT and mutants in their secondary structure. The non-amyloidogenic R555W was more sensitive to pH compared to the WT and amyloidogenic H572R. The WT (Fig. 1c) and H572R (Fig. 1e) remained unchanged under neutral and acidic pH. The R555W mutant displayed an increase in the CD intensity at 222 nm and 207 nm with decrease in pH (Fig. 1d). CD intensities at 222 nm at varying pH values (Fig. 1f) showed that R555W, was more sensitive to pH and the pH-response of H572R was similar to the WT protein. We also incubated the mutants in acidic pH for 1 week and followed their aggregation/oligomerization by ThT fluorescence (Supplementary Fig. S1). However, no significant conversion was observed as seen from the corresponding CD spectra for WT and H572R. At pH 2.75, R555W showed a partial conversion to β-sheet. Compared to the amyloid fibril peptide pN622K, almost no increase in fluorescence was observed for WT and R555W. The slight increase in fluorescence corresponding to pH 2.75 and pH 3.25 for H572R did not show a corresponding conversion in the CD spectra.

### Effect pH on thermal denaturation of WT and mutants

**Figure 2:**
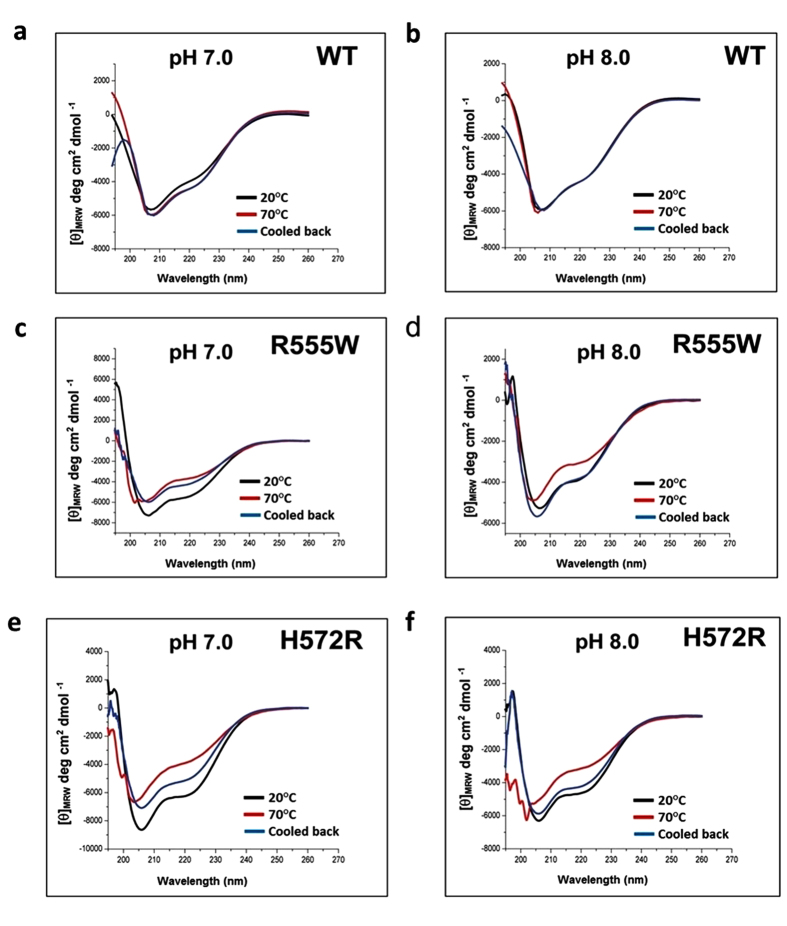
Thermal denaturation of the 4th_FAS1 domains of the WT and mutants at neutral and basic pH. (**a**,**b**) Far UV CD spectra of the 4^th^_FAS1 domain of WT at pH 7.0 and pH 8.0 before heating (black), after heating to 70 °C (red) and cooling back to 20 °C (blue). (**c,d**) Far UV CD spectra of the 4^th^_FAS1 domains of R555W at pH 7 (**c**) and pH 8 (**d**) before heating (black) and after heating to 70 °C (red) and cooling back to 20 °C (blue). (**e**,**f**) Far UV CD spectra of the 4^th^_FAS1 domains of H572R a pH 7.0 (**e**) and pH 8.0 (**f**) before heating (black) and after heating to 70 °C (red) and cooling back to 20 °C (blue). The WT and the mutants did not display any significant changes in structure at pH 7.0 and pH 8.0.

**Figure 3:**
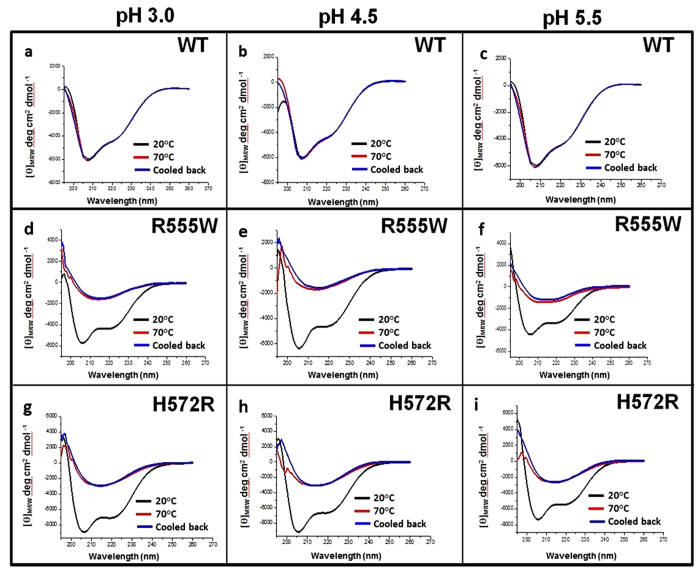
Thermal denaturation of the 4^th^_FAS1 domains of the WT and mutants at acidic pH. (**a**–**c**) Far UV CD spectra of the 4^th^_FAS1 domain of WT at pH 3.0 (**a**), pH 4.5 (**b**) and pH 5.5 (**c**) before heating (black) and after heating to 70 °C (red) and cooling back to 20 °C (blue). (**d**–**f**) Far UV CD spectra of the 4^th^_FAS1 domain of R555W at pH 3 (**d**), pH 4.5 (**e**) and pH 5.5 (**f**) before heating (black) and after heating to 70 °C (red) and cooling back to 20 °C (blue). (**g**–**i**) Far UV CD spectra of the 4^th^_FAS1 domain of H572R at pH 3.0 (**g**), pH 4.5 (**h**) and pH 5.5 (**i**) before heating (black) and after heating to 70 °C (red) and cooling back to 20 °C (blue). While the WT did not show any changes in structure, both the mutants displayed a very clear transition to β-sheet under acidic conditions.

**Figure 4:**
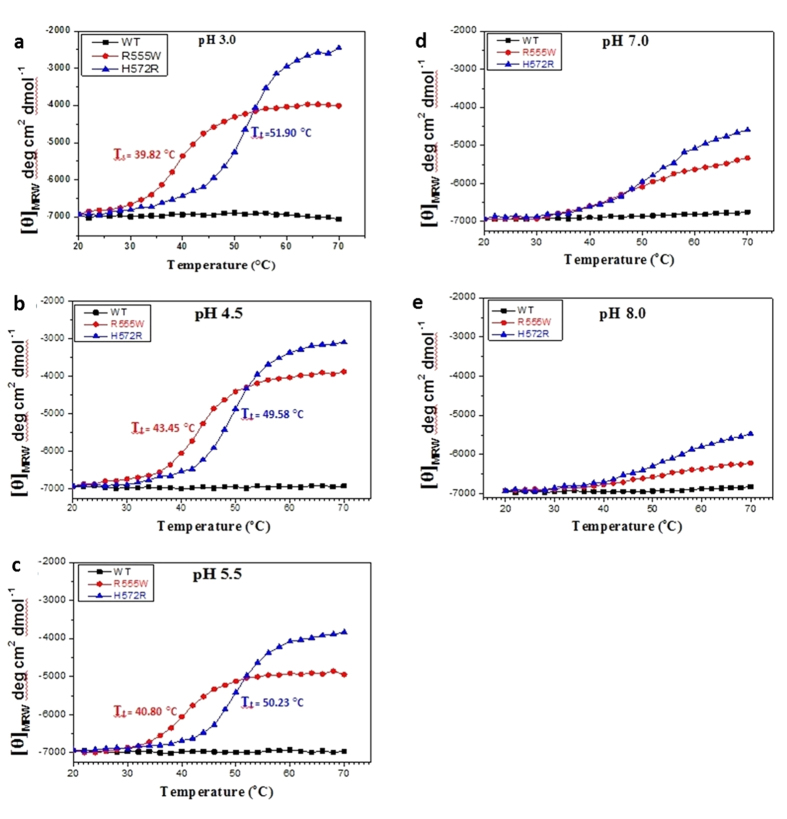
Difference in thermal denaturation induced transition between non-amyloidogenic and amyloidogenic mutants at acidic pH. (**a**–**e**) Variable temperature CD curves at 222 nm of WT (black), R555W (red) and H572R (blue) proteins heated from 20 °C to 70 °C at various pH (3.0 [**a**], 4.5 [**b**], 5.5 [**c**], 7.0 [**d**] and 8.0 [**e**]) and the CD intensities at 222 nm were plotted as a function of temperature. The baseline subtracted curves of the WT (black), R555W (red) and H572R (blue) proteins show that while there was no transition observed in the WT in all the conditions as observed from the unchanged straight line in black, little or no changes were seen in pH 7 and pH 8 for the mutants. However, clear transitions to β-sheet were observed at acidic pH (pH 3, pH 4.5 and pH 5.5) for both the mutants. In all cases, we observe transition (Tt) is higher for R555W compared to H572R. A clear shift in their thermal denaturation curves between the mutants at acidic pH (pH 3.0, 4.5 and 5.5) is observed. A difference in T_t_ of 5–12 °C is observed at various pH conditions.

Conformational transition of the domains undergoing thermal denaturation was examined by heating them from 20 °C to 70 °C, at neutral and acidic pH conditions. At pH 7 and pH 8, for both the mutants and the WT, no well-defined transition was observed with increasing temperatures (Fig. 2a–f). For both the mutants, the amplitude of the negative minima at 222 nm decreased upon heating (Fig. 2c–f) and a weak hysteresis was observed when cooled. With acidic pH, while the WT showed no apparent changes in the secondary structure with increasing temperature (Fig. 3a–c), both the mutants displayed a clear transition from monomeric α/β-structure to β-sheet (Fig. 3d–i). Single wavelength scan at 222 nm indicated a clear sigmoidal transition for both mutants under acidic conditions when compared to the WT, which was unperturbed by the changes in pH and temperature (Fig. 4a–e). Thermal denaturation experiments were also done by heating the domains from 20 °C to 90 °C (Supplementary Fig. S2). Though previous studies have observed denaturation of WT above 60 °C 32, we did not observe any conformational transitions to β-sheet even after heating to 90 °C. The mid-point of the normalized sigmoidal curve defines the transition temperature (T_t_) wherein the conversion of a monomeric α/β-structure to the β-structured oligomers was observed. The transition for the domains at different pH conditions was similar to the samples heated to 70 °C and the T_t_ lied between 35–58 °C. Hence all the subsequent thermal denaturation experiments were performed by heating the domains upto 70 °C. The non-amyloidogenic R555W showed a marked sensitivity to pH and displayed a higher thermal instability compared to the H572R. Though H572R mutant displayed a clear pH-dependent conversion to β-structure when heated, the T_t_ was higher than R555W. A difference in T_t_ of 5–12 °C is observed at various pH conditions. Mild precipitation was observed in the samples after heating, which correlates with the change in intensities observed in the CD spectra. When the concentration of the sample was increased (from 0.6 mg/ml to 1.2 mg/ml~75 μM), the turbidity and precipitation increases.

### Urea denaturation of non-amyloidogenic R555W

**Figure 5:**
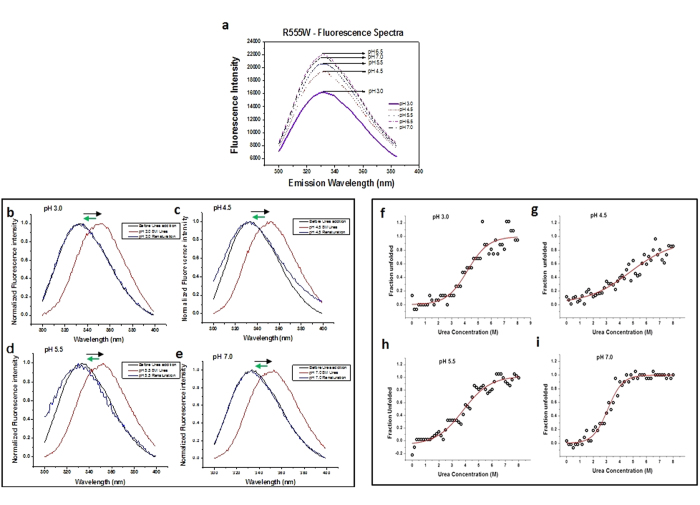
pH sensitivity and stability of the non-amyloidogenic (R555W) phenotype. (**a**) Fluorescence emission spectra of R555W with decrease in pH. There was a clear decrease in emission maximum at 332 nm with decrease in pH (indicated by the black arrow). (**b**–**e**) Fluorescence emission spectra of R555W showing the reversibility to folded state after removal of urea. The R555W mutant was incubated with increasing concentrations of urea from 0.25 M to 8 M at various acidic conditions (pH 3, pH 4.5, pH 5.5) and pH 7 and the emission fluorescence before and after urea incubation was measured. The emission spectra before urea incubation 332 nm (black), after incubating with 8 M urea (red) and after removing urea by buffer exchange (blue). Unfolding of the protein is seen by the shifting of peaks (black arrow) from 332 nm to ~ 352 nm. The refolding of the protein after removal of urea is seen by the return of the emission maximum to ~332 nm (green arrow). (**f**–**i**) Investigation of the stability the non-amyloidogenic phenotype using urea denaturation studies. The R555W mutant was incubated with increasing concentrations of Urea from 0.25 M to 8 M at various acidic pH (pH 3, pH 4.5, pH 5.5) and pH 7, and the emission fluorescence was measured. The denaturation plots of ‘fraction unfolded vs urea concentration’ were plotted and fit into a two state model, with the parameters calculated as described in the methods section.

The tryptophan residue in R555W allowed us to measure the emission fluorescence. Examination of the emission fluorescence at ~332 nm of R555W in acidic pH showed a significant decrease in emission intensity with decreasing pH, however the emission maxima remained unchanged (Fig. 5a). To obtain a better insight into the effect of pH on R555W, we monitored the urea-induced unfolding of R555W. Increasing the urea concentration progressively shifts the emission maxima (~332 nm) to longer wavelengths (~352 nm), suggesting a clear transition from folded to unfolded conformations (5b–e). To confirm refolding of unfolded R555W, the unfolded protein at different pH conditions (pH 3.0, pH 4.5, pH 5.5 and pH 7.0) was diluted appropriately and emission spectra were recorded. The emission maxima (λ_max_) plotted with the unfolded and refolded domains were superimposable (Supplementary Fig. S3). (Fig. 5b–e). A clear reversal in fluorescence maxima from ~352 nm to ~332 nm was observed thereby allowing us to estimate the thermodynamic stability of the mutant protein in various pH. Figure 5f–i shows urea denaturation curves plotted as ‘fraction unfolded (y_U_) *vs* increasing urea concentrations’ as monitored by the changes in emission maxima (Δλ_max_) at various pH values (Supplementary Fig. S3) for the R555W mutant. The thermodynamic parameters derived from urea denaturation are shown in the table (Table 2). A significant decrease in free energy of unfolding from 13.8 ± 0.8 kJ/mole to 7.108 ± 1.5 kJ/mole was observed from pH 7.0 to pH 4.5 for the R555W mutant. However, for the H572R mutant, fluorescence studies could not be performed because of the absence of a tryptophan residue.

### Characterization of the β-oligomers of amyloidogenic and non-amyloidogenic mutants

The amyloidogenic and non-amyloidogenic mutants displayed a clear transition to an all β-sheet conformation when heated under acidic conditions. Conversion to an all β-sheet conformation could indicate β-oligomer formation^17^. A detailed investigation of the β-oligomers from two mutants was therefore performed to validate and characterize the proposed β-oligomers.

### Confirmation of β-oligomers formed by the mutants and characterization of the β-oligomers by TEM and DLS

**Figure 6:**
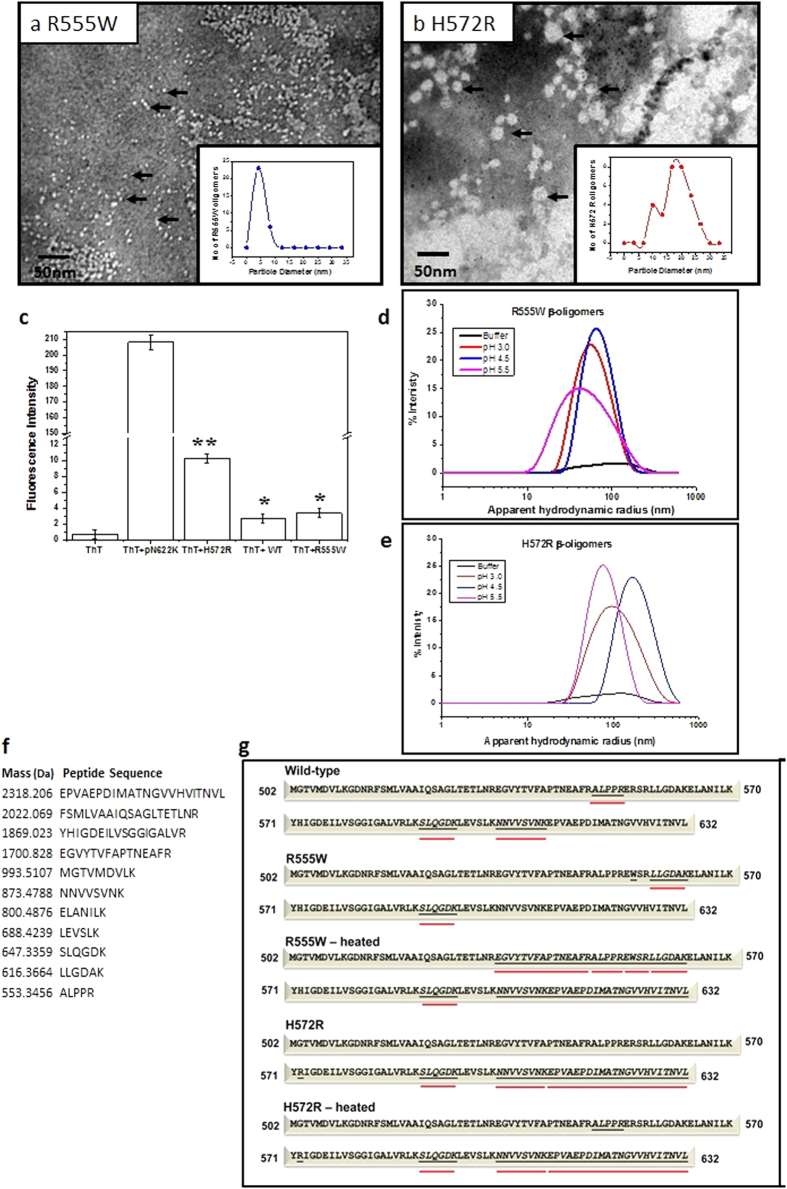
Characterization amyloidogenic and non-amyloidogenic β-oligomers. (**a**–**b**) Transmission Electron Microscopy. TEM images of the β-oligomers of the 4^th^_FAS1 domains of R555W (**a**) and H572R (**b**) mutants were acquired with a JEOL JEM-1010 transmission electron microscope using Digital Micrograph™ 1.81.78 for GMS 1.8.0. The β-oligomers of the amyloidogenic phenotype were larger measuring between 10–40 nm, mean size ~19.1 nm ± 4.9 nm (**a**) compared to the non-amyloidogenic β-oligomers that measured 4–8 nm, with a mean size ~5.1 nm ± 1.79 nm (**b**). Inset figures – particle size distribution of the β-oligomers. While the amyloidogenic β-oligomers were larger and displayed rugged edges and varying diameters, the non-amyloidogenic β-oligomers were smaller in size with smoother edges and were more homogenous. (**c**) ThT assay. Emission fluorescence intensities at 485 nm recorded after incubating the WT TGFβIp and the β-oligomers of R555W and H572R with ThT dye. An amyloid forming peptide (611-633aa - pN622K) of TGFβIp was used as the positive control. The β-oligomers of the amyloidogenic mutant H572R showed significant fluorescence intensity (**P < 0.01) almost 3 times more fluorescence compared to the non-amyloidogenic R555W. (**d**) DLS. %intensity plots plotted against the apparent hydrodynamic radii (R_H_). The R_H_ values calculated from the distribution curves for R555W (~39.58 nm|_pH 3.0_, ~51.9 nm|_pH 4.5_ and ~68.2 nm|_pH 5.5_) and H572R (~89.95 nm|_pH 3.0_, ~69 nm|_pH 4.5_ and ~155 nm|_pH 5.5_) show that H572R b-oligomers are larger in size and slightly more heterogeneous. (**e**,**f**) Mass Spectrometric analyses - Identification of the aggregation hotspots in the mutants and β-oligomers. (**d**) Peptide map generated following the insilico trypsin digestion of TGFβIp displaying a series of peptides. (**e**) The β-oligomers formed from the mutant 4^th^_FAS1 domains were digested with trypsin and the resulting fragments were analyzed by LC–MS/MS. The regions inaccessible for trypsin digestion have been underlined in red. It is clearly seen that the non-amyloidogenic R555W shows more regions inaccessible for trypsin digestion.

TEM examination of the β-oligomers revealed that while no particles were visible for the WT (data not shown), both the mutants displayed particles validating our proposal. The β-oligomers of the H572R and R555W displayed varying sizes and morphologies (Fig. 6a,b). The β-oligomers of the non-amyloidogenic R555W were homogeneous, displayed smoother edges and measured ~4–8 nm (mean diameter ~5.1 ± 1.79 nm) (Fig. 6a). The β-oligomers of the amyloidogenic H572R were more heterogeneous, displayed rugged edges and were larger, measuring 10–40 nm (mean diameter ~19.1 ± 4.9 nm) (Fig. 6b). The β-oligomers formed from the amyloidogenic and the non-amyloidogenic phenotypes are distinctly different from each other.

Dynamic light scattering (DLS) allows the examination of apparent hydrodynamic radius (R_H_) of a protein in solution^33^,^34^. DLS analysis on the β-oligomers prepared under acidic conditions show that H572R β-oligomers exhibit large variations in their R_H_ (~89.95 nm|_pH 3.0_, ~69 nm|_pH 4.5_ and ~155 nm|_pH 5.5_), while R555W β -oligomers are more uniform and almost similar across various acidic pH conditions (~39.58 nm|_pH 3.0_, ~51.9 nm|_pH 4.5_ and ~68.2 nm|_pH 5.5_). The distribution curves from the % intensity plots also show the homogeneity of the non-amyloidogenic R555W, and relative heterogeneity of the amyloidogenic H572R. This is in conjunction with the results obtained from TEM, where we see more homogenous and smaller β-oligomers from the non-amyloidogenic R555W mutant and heterogeneous and relatively larger β-oligomers from the amyloidogenic H572R mutant.

### The β-oligomers of the amyloidogenic H572R shows stronger binding to Thioflavin T (ThT)

Amyloid fibrils bind to the dye ThT and display an emission fluorescence at 485 nm^35^,^36^. In Alzheimer’s disease, ThT binds to Aβ-oligomers themselves, and has been proposed for early diagnosis^37^. We wanted to test if the TGFβIp β-oligomers bind to ThT. The amyloid forming TGFβIp peptide pN622K (pN622K^611–633^) displayed a high fluorescence intensity when bound to ThT (Fig. 6c). The fluorescence displayed by WT was comparable to the background fluorescence. Relatively, the β-oligomers of H572R showed significant fluorescence on binding to ThT (**P < 0.01) and atleast ~3 times more fluorescence compared to R555W. While the fluorescence intensities were much lower compared to the fibril forming peptide, it was significant that the β-oligomers of the amyloidogenic H572R were able to bind to ThT and this could aid further characterization of the β-oligomers.

### The mutants display differences in their ‘aggregation hotspots’

To determine the regions with high aggregation propensities or ‘aggregation hotspots’ within the mutants and the β-oligomers, the domains were digested with trypsin and the resulting peptides were examined using LC-MS/MS. The peptide map generated (Supplementary data) following the insilico trypsin digestion displayed a series of peptides (Fig. 6d) formed following trypsin digestion. We aimed to identify the regions that were probably buried within the β-oligomers and hence resisted trypsin digestion. For WT, three short peptides (^549^ALPPR^553^, ^591^SLQGDK^596 603^NNVVSVNK^610^) were not detectable after tryptic digestions. For R555W, two short peptides (^558^LLGDAK^563^, ^591^SLQGDK^596^ were absent. However, for the H572R, peptides in the region E611-L632 were not observed in addition to a short peptide (^591^SLQGDK^596^) that was absent in the WT and R555W. Mapping the generated peptides to the 4^th^_FAS1 domain displayed interesting results (Fig. 6e). The entire C-terminal region encompassing the residues ^603^NNVVSVNK^610^ and ^611^EPVAEPDIMATNGVVHVITNVL^632^ peptides was absent in the H572R native protein and β-oligomers. This region was intact in WT and R555W. A long stretch of residues between E^534^ and K^563^ containing the peptides ^534^EGVYTVFAPTNEAFR^548^, ^549^ALPPR^553^, ^554^EWSR^557^, ^558^LLGDAK^563^ was not observed in R555W β-oligomers. The peptide ^591^SLQGDK^596^ was absent in all the proteins. The segment ^549^ALPPR^553^ was absent in H572R β-oligomers. The β-oligomers of R555W, displayed a region extending between E^534^ and A^562^ that could possibly be buried. It is interesting to note that the mutation R555W resides within this region. The β-oligomers of H572R did not reveal such a region within the domain. It is likely that different regions of the 4^th^_FAS1 domain may be involved in the formation and stabilization of these β-oligomers.

### The β-oligomers remained stable at physiological conditions

**Figure 7:**
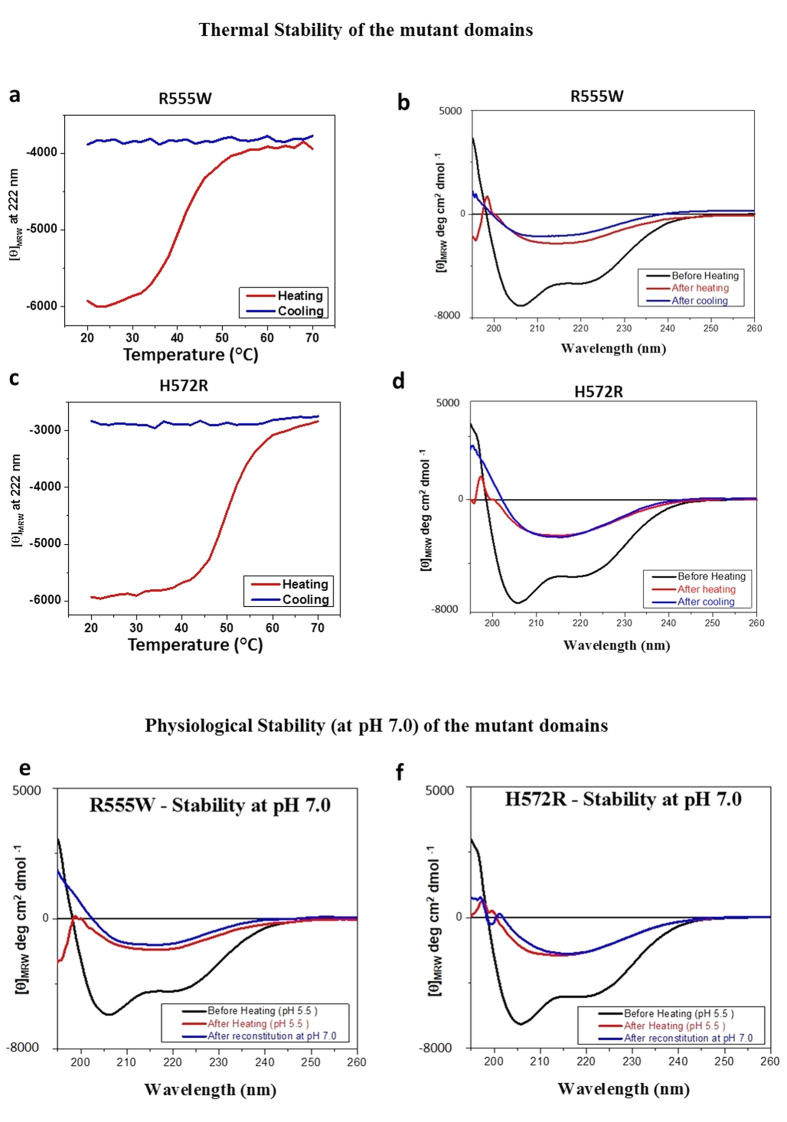
Stability of the β-oligomers at physiological conditions. (**a**–**d**) Thermal stability. Variable temperature CD values at 222 nm of the 4^th^_FAS1 domains of R555W (**a**) and H572R (**c**) in buffer at pH 5.5 when heated from 20 °C to 70 °C. Far UV CD spectra of the 4^th^_FAS1 domains of R555W (**b**) and H572R (**d**) after heating (red) to 70 °C and cooling (blue) back to 20 °C. (**e**,**f**) Stability at physiological pH. Far UV CD spectra of the 4^th^_FAS1 domains (black) of R555W (**e**) and H572R (**f**) at pH 5.5 under native conditions (black), after heating to 70 °C (red) and after reconstituting in buffer at pH 7.0 (blue) after incubation at room temperature for 4 weeks.

For studying the thermal stability of the β-oligomers of the R555W (Fig. 7a,b) and H572R (Fig. 7c,d) the mutant domains at pH 5.5 were heated to 70 °C and cooled back to 20 °C. The β-oligomers remained in their β-sheet conformation at 20 °C showing no reversibility to the native α/β-conformation. To test their stabilities at physiological pH, the β-oligomers formed at pH 5.5 were reconstituted to pH 7 and examined (Fig. 7e,f). The samples were also incubated for 4 weeks at pH 7 and the CD spectra were recorded. In both cases, the β-oligomers remained in their stable β-sheet conformation. This demonstrates that the β-oligomers were stable to thermal changes and at physiological conditions allowing for further examination.

### Cytotoxicity of β-oligomers

**Figure 8:**
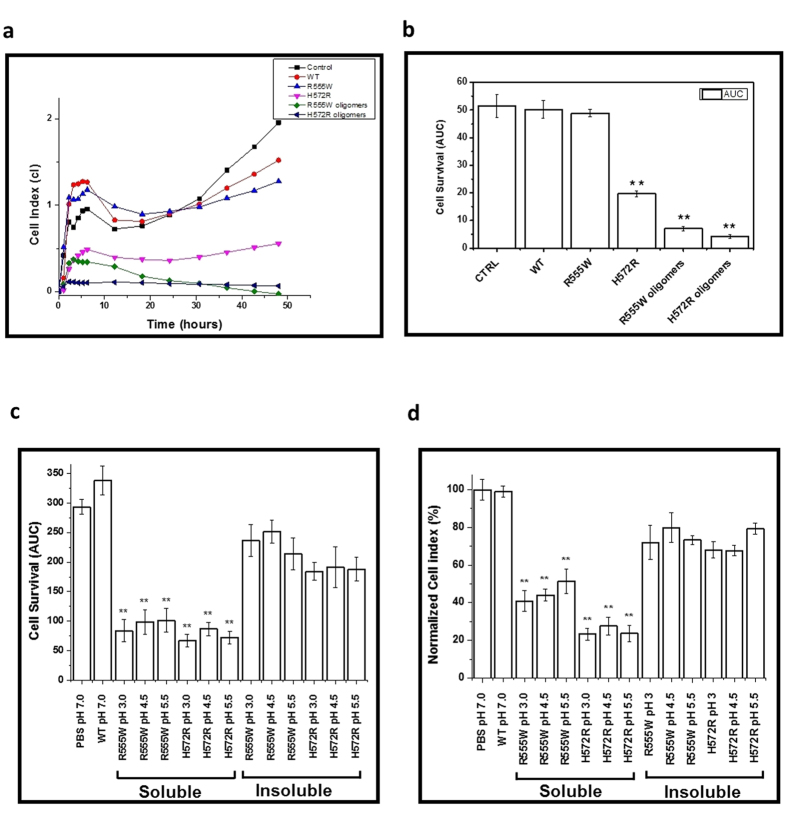
Cytotoxicity. (**a**) The growth and proliferation of the pHCSF cells following the treatment with the β-oligomers and the mutant 4^th^_FAS1 domains monitored for 48 hours in an xCELLigence system. (**b**) The cell survival after treatment for 24 hours plotted as area under the curve (AUC). While the WT and R555W show low cytotoxicity, almost similar to the media control, the amyloidogenic H572R mutant was cytotoxic compared to the control (**P < 0.01). Interestingly, the β-oligomers of both the amyloidogenic and non-amyloidogenic mutants showed high cytotoxicity (**P < 0.01) with the approximately 7 times more for the non-amyloidogenic mutant and 12 times more for the amyloidogenic mutant. To obtain detailed information on the cytotoxicity of the soluble and insoluble fractions, β-oligomers of the two mutants were prepared at various acidic pH conditions (pH 3.0, pH 4.5, pH 5.5) and their cytotoxicity were examined on pHCSFs from 3 different donors (n = 3) using xCELLigence (**c**) and MTT assays (**d**). (**c**) xCELLigence assay. The WT shows low cytotoxicity, almost similar to the control. The soluble β-oligomers of both the mutants showed high cytotoxicity (**P < 0.01) compared to the controls. The insoluble β-oligomers were relatively less cytotoxic compared to the soluble β-oligomers. (**d**) MTT assay. The cytotoxicity of the β-oligomers was also tested using an MTT assay. Similar to xCELLigence, we could see that soluble β-oligomers derived from both the mutants displayed potent cytotoxic effect (**P < 0.01) compared to the controls and insoluble aggregates. Though the insoluble oligomers were relatively less cytotoxic than the soluble oligomers, they displayed cytotoxicity compared to the controls.

The toxicity of the β-oligomers on primary human corneal stromal fibroblast (pHCSF) cells was monitored using xCELLigence system. The WT domain did not interfere with the cell adhesion and proliferation and exhibited little or no toxic effect on the seeded cells (Fig. 8a). R555W also displayed no cytotoxicity. H572R, however, displayed higher cytotoxicity (**P < 0.01) on the fibroblasts compared to R555W (Fig. 8b). Interestingly, β-oligomers from both R555W and H572R were cytotoxic (**P < 0.01). While, the β-oligomers of R555W decreased the cell proliferation and the cytotoxic effect was visible after 12 hours, the β-oligomers of H572R displayed the maximum cytotoxic effect as no proliferation was observed and the xCELLigence showed minimum cell index. These results suggest that amyloidogenic mutant displayed significant cytotoxic effect both in the native as well as in the β-oligomeric forms whereas the non-amyloidogenic mutant was cytotoxic in the β-oligomeric form only. To obtain detailed information on the cytotoxicities, we prepared the β-oligomers of the two mutants at various acidic pH (pH 3.0, pH 4.5, pH 5.5). We also included the insoluble aggregates obtained after centrifuging the mildly precipitated samples. When we examined this precipitates using CD, we found similar β-sheet curves as we found for the soluble oligomers (data not shown). Hence, we investigated these “insoluble aggregates” along with the soluble β-oligomers. The populations were reconstituted to pH 7 using buffer exchange. pHCSFs from 3 different donors (n = 3) were treated with the β-oligomers and examined (Fig. 8c). The WT shows almost no cytotoxicity, similar to the control. The soluble β-oligomers derived from both the mutants at various acidic pH conditions displayed potent cytotoxic effect (**P < 0.01) compared to the insoluble aggregates and controls. The insoluble aggregates were also cytotoxic (*P < 0.05) but were relatively lesser compared to the soluble β-oligomers. The results were also validated using an MTT assay (Fig. 8d). Similar to xCELLigence, we could see that soluble β-oligomers derived from both the mutants displayed potent cytotoxic effect (**P < 0.01) compared to the controls and insoluble aggregates.

## Discussion

The most significant aspect of the TGFβIp associated corneal dystrophies is that the single amino acid substitutions in the mutants are responsible for clinically distinct phenotypes. Here, we examined the properties of two mutants, the amyloidogenic H572R^22^,^23^, and non-amyloidogenic R555W^24^,^25^,^26^. Previous attempts to study the differences between the WT and the mutants at physiological conditions have revealed minimal information on the mechanism of their aggregation^38^. To elucidate the inherent variations between these mutants, we examined the effects of various biophysical and biochemical factors like temperature and pH on them. In amyloid forming proteins such as β-microglobulin^39^, thermal unfolding leads to formation of β-oligomers. Results from our thermal denaturation studies show clear differences between both the phenotypes and the WT protein at acidic pH.

Our results suggest that changes in the physiological pH can influence the biochemical and biophysical characteristics of TGFβIp mutants. A clear conversion from α/β-structure to β-sheet was observed when the mutants were heated under acidic conditions (<pH 5.5). TEM and DLS studies confirmed the formation of β-oligomers by both phenotypes that remain irreversible and stable at physiological pH and temperature. For the first time we have shown that both the amyloidogenic and non-amyloidogenic phenotypes displayed marked differences in their sensitivities to pH and β-oligomer formation. The two β-oligomers of different phenotypes exhibit variations in their morphologies, sizes, thermal and chemical stabilities, aggregation patterns and cytotoxicity.

Supporting our results that acidic pH could play an important role in the aggregation of proteins *in vivo*, some recent studies have provided insights on the possible influence of acidic pH on the aggregation of proteins^40^,^41^,^42^. Pfefferkorn *et al.*, have reported that Pmel 17, a functional amyloid involved in the structural scaffolding for melanin deposition in human skin and eyes, displays a strong tendency to form fibrils at acidic pH 5.0 43. The Pmel 17 fibrils are formed inside the melanosomes, organelles related to lysosomes that have acidic environments similar to lysosomes. Also, in studies involving TGFβIp associated dystrophies, Kim *et al.*, have reported^31^ that the R124H mutation in TGFβIp disrupts its binding to periostin and causes TGFβIp to localize in lysosomes. It is well known that the lysosomal environment is highly acidic. While the mutation affects the binding of TGFβIp to its functional partners, the localization of TGFβIp mutants to the lysosomes with acidic pH could possibly induce the initiation of β-oligomer formation. While the 4^th^_FAS1 domain is not known to bind to periostin, it is still distributed to lysosomes by the R124H mutation^31^. It is known that the proteolytic fragments in patient samples include the 4^th^_FAS1 domain along with the 1^st^ FAS1 domain^44^. Acidic environments could probably induce the conversion to β-sheet in the 4^th^_FAS1 domain. We also speculate that under acidic pH, binding of TGFβIp to periostin might be disrupted possibly due to the formation of β-oligomers in the presence of other interacting factors. TGFβIp is known to interact with several extracellular matrix proteins like various types of collagen, biglycan, decorin and fibronectin^31^ and it is possible that the acidic pH could probably have a disruptive effect on the binding of the mutants to other ECM components. Therefore, it is reasonable to suggest that acidic environments could play an important role in the aggregation of dystrophic mutants. At physiological conditions (pH 7.0), there were no perturbations in structure from the native conformation. Under acidic conditions (pH 5.5 and below), we were able to show the ability of the mutants to form β-oligomers. Any change in the stromal environment causing a lowering of pH to about 1.5 units could probably be a trigger to initiate the formation of β-oligomers. Physiological events like lysosomal membrane disruption that can alter the pH values of the intracellular and extracellular microenvironment by a pH reduction of ~1.5 (pH 7 →pH 5.5) during cellular processes such as wound-healing and phagocytosis^45^,^46^,^47^ could possibly lead to conversion to β-sheet and possibly formation of β-oligomers.

We performed long-term incubation of the WT and mutants in acidic pH conditions and followed their possible aggregation/oligomerization using ThT fluorescence and CD. Both the mutants did not display any aggregation/oligomerization over 1 week (Supplementary Fig. S1). We also performed seeded fibrillation experiments using β-oligomers as seeds and incubated them for 2 weeks. However, we did not observe any significant increase in ThT fluorescence (Supplementary Fig. S4) within the period. There could be other cellular factors and interacting molecules involved directly or indirectly with the aggregation that need to be studied along with the acidic pH at physiological temperature to induce aggregation/oligomerization. Prolonged incubation under these conditions could probably induce the formation of oligomerization in both the cases. Also, in diseases conditions, patients harbor the mutations from birth, but the ages of onset range from few years to few decades. While we emphasize the significance of acidic pH in the possible aggregation and β-oligomer formation of the 4^th^_FAS1 domain, we certainly believe that more detailed *in vivo* investigation is needed to delineate the aggregation mechanism and the molecules.

At neutral pH, we have seen that the R555W mutant is more stable than the WT. This was also seen in the stability experiments by Runager *et al.*^48^. However, from our results we see that the non-amyloidogenic R555W displays high susceptibility to acidic pH. In R555W, the mutation (R→W) causes an overall reduction in net charge from −0.6 to −1.6 at pH 7.0 (17, Fig. 1c). At pH 5.5, the charge reduces from 1.9 to 0.9 17. The substitution of the hydrophobic tryptophan (−2.13) in place of the polar arginine (3.95) also increases the hydrophobicity, thereby altering the solubility and possible folding of the mutant protein The reduction in charge coupled with the increase in hydrophobicity might influence the sensitivity of R555W to acidic pH. In H572R, the histidine residue is replaced by arginine residue. Histidine has a neutral charge at pH 7 and positive charge at acidic pH (pH < 6.0). While there is an increase in the overall charge from −0.6 to 0.2 by the substitution of arginine at pH 7, there is almost no change in the overall net charge for the H572R mutant under acidic conditions (1.9 to 2.0 at pH 5.5). This may explain the relatively higher stability of the H572R mutation at lower pH when compared to R555W. One of the important observations is the relative instability or sensitivity of R555W (pI 6.32) to pH 4.5. Our results from pH incubation studies, urea denaturation studies show that around pH 4.5, there is a decrease in stability for R555W. Further characterization of R555W at pH 4.5 might provide more details on the significance of this pH.

It was also important to understand relative instability of R555W from a structural perspective. In recent studies, (Runager *et al.*^48^ and Underhaug *et al.*^49^) have observed that the R555W mutant is more stable than the WT^48^ and the 4^th^_FAS1 domain of R555W has a more compact structure^49^ at pH 7. Underhaug *et al.* also observed that the α3′ helix, harboring the Arg555 residue, shows an increase in connected surface of negative charge in the R555W mutant compared to the WT which might directly influence its charged molecular interactions. They also found that in the presence of acidic conditions (10% TFE) R555W mutant exhibits higher aggregation propensity compared to the WT. The increased concentration of negative charges on the molecular surface of the R555W 4^th^_FAS1 domain could possibly explain its sensitivity to acidic pH and also its instability at pH 4.5.

Various protein aggregation diseases involve β-oligomeric intermediates^50^,^51^,^52^. These β-oligomers could potentially act as seeds for the formation of amyloid fibrils^53^,^54^, and hence understanding the stability of β-oligomers becomes vital. In our study, the β-oligomers from both the phenotypes formed at pH 5.5 remain stable at physiological conditions for more than 4 weeks. Interestingly, the two β-oligomers seem to adopt different modes of oligomerization and also exhibit differences in their sizes, ThT-binding and cytotoxicity. Pfefferkorn *et al.*^43^, have shown in Pmel 17 that one of the first steps in fibril formation is the formation of β-oligomers that appear as large laterally associated small fibrillar species. At pH 4.5–5.0, a mix of amorphous and fibrillar structures were observed. Consistent with this, we have seen formation of β-oligomers that appear as spherical structures with rugged edges of different sizes using TEM. The amyloidogenic H572R displays larger particles compared to the non-amyloidogenic R555W. Previous studies in protein aggregation disorders like Parkinson’s syndrome have shown that the β-oligomers are more toxic than the amyloid fibrils themselves^53^,^54^,^55^. This has however not been studied in *TGFBI*-associated corneal dystrophies. When we assessed the cytotoxicity of the β-oligomers in pHCSF, β-oligomers from amyloidogenic H572R were more cytotoxic than the non-amyloidogenic R555W. In serum amyloid A (SAA) peptides^55^, the pathogenic and non-pathogenic strains appear as two distinct oligomeric populations (SAA1.1 and SAA2.2). While the non-pathogenic SAA2.2 forms smaller oligomers and grows into braided fibrils, the pathogenic SAA1.1 forms larger oligomers and grows into straight fibrils. Similar to this, the larger β-oligomers from amyloidogenic H572R are more cytotoxic compared to the smaller β-oligomers of the non-amyloidogenic R555W.

We have seen that the soluble β-oligomers of both mutants were more cytotoxic compared to the insoluble aggregates. This leads us to believe that while the soluble β-oligomers are responsible for cell death in the corneal dystrophies, the insoluble aggregates are more responsible for scattering of light and hence loss of visual acuity. This could also mean that cytotoxicity due to β-oligomer formation precedes the loss of sight due to accumulation of protein aggregates. This provides us an important understanding in studying the modes of treatments of corneal dystrophies wherein, the hastening of the intermediary soluble β-oligomers to form insoluble and stable higher order aggregates such as fibrils and granules, could minimize cell death.

In conclusion, these results suggest that changes in the pH may cause localized perturbations in the secondary structure of the mutant proteins and increase the propensity to form β-oligomers. We have shown that under similar biophysical and biochemical conditions, the different mutant phenotypes exhibit differences in morphology, size and cytotoxicity. However, we did not observe the conversion of β-oligomers into amyloid fibrils even after incubation for 4 weeks. The soluble β-oligomers exhibit higher cytotoxicity compared to the insoluble β-oligomers. The interaction of β-oligomers with other extracellular matrix proteins will shed light into the mechanism of amyloid formation in future studies.

## Methods

### Materials

The cDNA constructs of the 4^th^_FAS1 domains of the WT TGFβIp, the mutants R555W and H572R were bought from Genscript (Piscataway, NJ) in pUC57 vectors. *E.coli BL21(DE3)* expression competent *cells*, Ek/LIC cloning kit *and* pCDF-2 vector system were purchased from Novagen (Novagen (EMD), PA). High-performance Ni-Sepharose resin was purchased from GE Healthcare (GE healthcare Life Sciences, NJ). Amicon Centriprep filter units were purchased from EMD Millipore (EMD Millipore, MA). Ampicillin, streptomycin, isopropyl β-D-thiogalactopyranoside (IPTG) and Thioflavin T were purchased from Sigma-Aldrich (Sigma-Aldrich Inc., MO). Formvar-carbon coated nickel grids were bought from EMS (Electron Microscopy Sciences, PA).

### Cloning, Protein expression and purification

Recombinant 4^th^_FAS1 domains of the WT, H572R and R555W TGFβIp proteins were cloned, expressed and purified as described previously^21^. Briefly, the *TGFBI* genes were cloned in pCDF2 vectors and expressed in *E.coli* BL21DE3 cells. The proteins were purified by affinity chromatography using a manually prepared column with High Performance Ni-sepharose beads. Further purification was done using RP-HPLC (C4 column, Phenomenex) with a linear gradient of 50% acetonitrile in 0.01% trifluoroacetic acid at a flow rate of 3 ml/min. The purified proteins were lyophilized and stored in −20 °C.

### Far-UV Circular Dichroism spectropolarimetry

Far UV–CD spectra of the proteins were collected using a Jasco J-810 spectropolarimeter (Jasco Inc., Easton, MD) using a quartz cuvette with a path length of 0.1 cm (Hellma, Müllheim, Germany). Purified 4^th^_FAS1 WT and mutant TGFβIp proteins were reconstituted in buffers of varying pH (pH 3.0–8.0) to a final concentration of ~0.6 mg/ml (~37 μM) and their CD spectra were recorded.

For thermal denaturation experiments, the samples in buffers of varying pH (pH 3.0, pH 4.5, pH 5.5, pH 7.0 and pH 8.0) were heated from 20 °C to 70 °C at a rate of 1 °C/min using the inbuilt Peltier heating system. Thermal denaturation experiments were also performed by heating the samples from 20 °C–90 °C under the same conditions. The spectra were recorded from 260 nm to 190 nm with a step-size of 0.1 nm, at a scan rate of 50 nm/min for every 2 °C. Variable temperature (VT) scanning was done by heating the sample to 70 °C at a rate of 1 °C/min and measuring the ellipticity at 222 nm. Initial and final spectra before and after heating were recorded. The final spectrum was the average of three scans. The CD data were expressed as mean residual weight ellipticity (deg cm^2^ dmol^-1^). The mean residual weight (MRW) ellipticity ([θ]_MRW_) was estimated using the equation


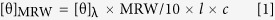


where [θ]_λ_ is the observed ellipticity, MRW is mean residual weight and defined as M/N−1 where M is the molecular mass and N is number of amino acid residues, l is the path length of the cuvette and c is concentration in mg/ml. The data obtained were fit into a two-state model using Origin 8.0 software and analysed using previously reported methods^56^,^57^,^58^.

For thermal stability experiments, the samples were heated to 70 °C and cooled back to 20 °C at a rate of 1 °C/min and the corresponding ellipticities at 222 nm were recorded. Far UV-CD spectra of the samples before heating, after heating to 70 °C and after cooling back to 20 °C were recorded. To test the effects of temperature ramping rates on the thermal unfolding, the mutant proteins were heated at pH 5.5, at various rates of thermal ramping (0.5 °C/min, 1 °C/min, 2 °C/min and 10 °C/min) and the CD spectra were recorded. VT curves were generated by measuring and plotting the CD intensity at 222 nm from 20 °C–70 °C.

For testing the stability of the β-oligomers at pH 7, the β-oligomers were formed by heating the mutants at pH 5.5, to 70 °C. Far UV-CD spectra after heating were recorded for the samples. The heated samples were centrifuged at 14,600 rpm (~20,000 g) and reconstituted in PBS buffer at pH 7.0 and kept at room temperature for 4 weeks and their CD spectra were recorded.

### Fluorescence spectroscopy

The 4^th^_FAS1 domain of R555W was incubated at various acidic conditions (pH 3, pH 4.5, pH 5.5 and pH 7.0) and the emission spectra from 300 to 400 nm were recorded following excitation at 280 nm for each sample using the Quanta Star spectrofluorimeter (Photon Technology International, NJ) using a 10 mm quartz cuvette. For urea denaturation studies, the R555W mutant protein (0.6 mg/ml – 37 μM) was treated with 0.25 M to 8 M urea for 16 hours at 4 °C. The intrinsic fluorescence corresponding to the tryptophan residue in R555W was measured as described above. To confirm the reversibility of the urea denatured proteins to their native conformation after removal of urea, the R555W protein was first incubated in buffers of different pH (pH 3.0, pH 4.5, pH 5.5 and pH 7.0) with 8 M urea for 24 hours. The unfolding of the proteins in the presence of 8 M urea was confirmed using fluorescence spectroscopy. These unfolded proteins were then reconstituted in buffers without urea by buffer exchange by centrifugation using Amicon Centriprep filter units. The emission peaks were measured. Reversibility studies were performed by diluting the R555W domain from 8 M urea to appropriate concentrations.

The denaturation data were fitted by non-linear least squares method, assuming a two state model^59^. To calculate the thermodynamics parameters from the chemical denaturation data, we first plotted the emission fluorescence spectra of the R555W mutant subjected to unfolding with 8 M urea. After confirming the shift in peaks from ~332 nm to ~ 352 nm, the differences in the wavelengths


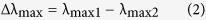


corresponding to the emission peaks were plotted against urea concentration to estimate the λ_U_ (unfolded) and λ_F_ (folded). The fraction-folded


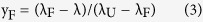


and fraction-unfolded


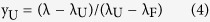


were calculated. The ΔG values were calculated by substituting values in the equation


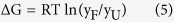


The resulting ΔG values from the linear range were plotted against urea concentration. The Y-intercept of the resulting straight line was taken as ΔG_H2O_ and the slope (m) was estimated.


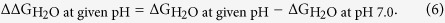


### Transmission Electron Microscopy (TEM)

TEM images of the β-oligomers prepared at pH 5.5 were acquired with a JEOL JEM-1010 transmission electron microscope using Digital Micrograph™ 1.81.78 for GMS 1.8.0 (Gatan, Pleasanton, CA) in the National University of Singapore Electron Microscopy facility. The samples were centrifuged and applied on Formvar-carbon coated nickel grids with bacitracin as the binding agent and negatively stained with 10% phosphotungstic acid (PTA). The samples were dried and observed at magnifications 8000–50000X at 80 kV. The size of the particles were estimated using the relation,


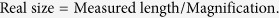


### Thioflavin T assay

The native and heated domains were treated with 30 μM thioflavin T (ThT) in PBS buffer at pH 5.5 in a Greiner 96-well flat bottom polystyrol microplate (Greiner, Frickenhausen Germany). The samples were excited at 445 nm and the resulting emission fluorescence at 485 nm was measured using a microplate reader (Tecan infinite M200 pro, Zanker Road, SJ). The emission fluorescence was monitored for 24 hours. A 20-amino acid long peptide (pN622K) from the 4^th^_FAS1 domain of TGFβIp with the mutation N622K (EPVAEPDIMATKGVVHVITNVLQ) capable of forming amyloid fibrils was used as positive control.

The native and heated 4^th^_FAS1 domains of the WT, R555W and H572R were incubated in buffers of different pH (pH 2.75, pH 3.25, pH 4.0, pH 4.5, pH 5.5 and pH 7.0) along with 30 μM thioflavin T (ThT) and their corresponding changes in fluorescence intensity at 485 nm were measured for a period of 7 days using the Tecan microplate reader. The N622K peptide was used a positive control.

Seeded fibrillation studies were performed by treating the domains with their respective β-oligomers prepared at pH 5.5 using thermal denaturation as described above. The samples were shaken in an orbital shaker at 37 °C at 180 rpm. The samples were taken after 2 weeks and mixed with ThT for a final concentration of 30 μM and the fluorescence intensities were measured as described before.

### Dynamic light scattering (DLS)

The β-oligomers were formed by heating the 4^th^_FAS1 domains of the R555W and H572R in buffers of different pH (pH 3.0, pH 4.5, pH 5.5) to 70 °C. The buffers were filtered using a 0.2 μM syringe filter before the thermal denaturation experiments. These prepared samples were examined using a DynaPro Plate ReaderII (Wyatt Technology, CA, US) equipped with a Peltier temperature controller. Each samples was measured 3 times and the average of the 3 scans is reported. The average and standard deviation values of the sizes corresponding to the peak of interest in each of these 3 distributions provides the apparent hydrodynamic radius and the experimental error for each samples respectively.

### Mass spectrometric analysis

The native and heated 4^th^_FAS1 domains of the WT, R555W and H572R were digested with trypsin. The tryptic-digested peptides were analyzed by LC–MS/MS [Ultimate 3000 nanoLC (Thermo Fisher Scientific/Dionex, Sunnyvale, CA), coupled with AB SCIEX Triple TOF TM 5600 mass spectrometer]. The peptide mixture was first desalted and pre-concentrated in a trap column (Acclaim PepMap 100 C18, 75 μm × 3 μm, 100 Å from Thermo Fisher Scientific/Dionex) for 3 min at a flow rate of 5 μl/min. After desalting, the system was switched into line with the reversed phase analytical capillary column (25 cm × 75 μm i.d. Acclaim PepMap RSLC C18, 2 μm, 100 Å, Thermo Fisher Scientific/Dionex, Sunnyvale, CA). A 35 min gradient was used at 300 nl/min. All data was acquired using information-dependent acquisition (IDA) mode with Analyst TF 1.5.1 software (AB Sciex, USA). Protein Pilot software (version 4.01, AB Sciex) was used to analyze the MS/MS data.

### Cytotoxicity

The xCELLigence system (ACEA, San Diego, CA, USA) was used to investigate the effects of the β-oligomers on pHCSF proliferation and cell death. Their E-plate 96 is incorporated with sensor arrays at the bottom of each well, which detects and translate cell attachment in the form of electronic impedance. A parameter termed cell index (CI) is derived which corresponds to the relative density and adherence strength of cells in each well^60^. pHCSF were seeded at a density of 5000 cells per well on an E-plate 96, in fibroblast media with 5% FBS. An initial concentration of ~37 μM (0.6 mg/ml) of the mutant protein was subjected to thermal denaturation at acidic pH to generate the β-oligomers. The β-oligomers were mixed with DMEM media at a ratio of 1:1 (~18 μM/0.3 mg/ml final concentration) and added to pHCSF cells. Culture was maintained for 48 hours in the xCELLigence RTCA SP Station, placed in an incubator at 37^o^  C with 5% CO_2_. No media change was done to reduce the fluctuations generated from removal of plate from RTCA system. The area under the curve (AUC) values were calculated from the cI values using the Mathematics module in OriginPro software and were used for further analysis.

To examine the cytotoxicities of the soluble and insoluble fractions in the β-oligomers, the β-oligomers of the two mutants prepared at various acidic pH conditions (pH 3.0, pH 4.5, pH 5.5) were were centrifuged at 14,600 rpm (~20,000 g) to separate the insoluble fractions. The insoluble β-aggregates in the pellets were reconstituted in PBS buffers of pH 7.0. For the soluble β-oligomers in the supernatant, buffer exchange by centrifugation using Amicon centriprep was done with PBS at pH 7.0. The soluble and insoluble populations were added to pHCSF cells from 3 donors (n = 3) and was monitored in an xCELLigence system. Cell survival assay using the xCELLigence system was performed as explained earlier. To test the cell viability, MTT assay was performed in parallel. The cells were incubated with the β-oligomers for 48 hours and treated with MTT (3-(4,5-dimethylthiazol-2-yl)-2,5-diphenyltetrazolium bromide) and incubated for 4 hours. The resulting color change was measured using the Tecan microplate reader (Tecan infinite M200 pro, Zanker Road, San Jose, USA).

### Statistics

All numeric data obtained were expressed as mean ± standard deviation. For both the ThT binding assays and cytotoxicity studies using xCELLigence, all the comparisons were done using one-way ANOVA followed by post-hoc Bonferroni test (SPSS Statistics 22.0, IBM, Chicago, IL) for multiple comparisons. For both the experiments, values were deemed to be significant (*) when a significance level with a *p*-value of less than 0.05 was achieved and very significant (**) when a significance level with a *p*-value of less than 0.01 was achieved.

## Additional Information

**How to cite this article**: Murugan, E. *et al.* pH Induced Conformational Transitions in the Transforming Growth Factor β-Induced Protein (TGFβIp) Associated Corneal Dystrophy Mutants. *Sci. Rep.* **6**, 23836; doi: 10.1038/srep23836 (2016).

## Supplementary Material

Supplementary Figures

Supplementary Data

## Figures and Tables

**Table 1:** Effects of mutation on the charge and hydrophobicity.

	WT	R555W	H572R
Charge of the mutation	–	R ( + 1) → W(0)	H (0) → R(+1) (at pH 7.0)
			H (+1) → R(+1) (at pH < 6.0)
Net Charge (at pH 7.0)	−0.6	−1.6	0.2
Net Charge (at pH 5.5)	1.9	0.9	2.0
Change in hydrophobicity due to the mutation (17)	–	3.95 → −2.13 (at pH 7.0 and pH < 6.0)	0.64 → 3.95 (at pH 7.0)
			2.87 → 3.95 (pH < 6.0)

The change in the individual charges and overall net charge in the mutants. The change in hydrophobicities were calculated using the equation described previously (17).

**Table 2:** Urea denaturation studies of the non-amyloidogenic phenotype.

pH	Δ 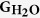 Z, kJ/mole	C_m_, M	m, kJ/mole/M	ΔΔGH_2_O, kJ/mole
3.0	8.417 ± 1.03	4.2 ± 0.15	3.14 ± 0.32	−5.383
4.5	7.108 ± 1.5	4.74 ± 0.91	1.85 ± 0.25	−6.692
5.5	11.28 ± 2.02	3.93 ± 0.21	2.78 ± 0.48	−2.52
7.0	13.8 ± 0.8	3.09 ± 0.1	3.8 ± 0.3	–

The R555W 4^th^ FAS1 domain was incubated with increasing concentrations of urea from 0.25 M-8 M at various acidic pH conditions and the emission fluorescence was measured. The free energies at various pH conditions were calculated and tabulated.

## References

[b1] Weiss J. S. *et al.* The IC3D classification of the corneal dystrophies. Cornea 27 (Suppl. 2) S1–S83 (2008). 19337156 10.1097/ICO.0b013e31817780fb PMC2866169

[b2] Klintworth G. K. Corneal dystrophies. Orphanet J. Rare Dis. 4, 7 (2009). 19236704 10.1186/1750-1172-4-7 PMC2695576

[b3] Surguchev A. & Surguchov A. Conformational diseases: looking into the eyes. Brain Res. Bull 81, 12–24 (2010). 19808079 10.1016/j.brainresbull.2009.09.015

[b4] Lakshminarayanan R. *et al.* Biochemical properties and aggregation propensity of transforming growth factor-induced protein (TGFβIp) and the amyloid forming mutants. Ocul Surf. 13(1), 9–25 (2015). 25557343 10.1016/j.jtos.2014.04.003

[b5] Kitahama S. *et al.* Expression of fibrillins and other microfibril-associated proteins in human bone and osteoblast-like cells. Bone 27, 61–67 (2000). 10865210 10.1016/s8756-3282(00)00292-1

[b6] Ferguson J. W. *et al.* The extracellular matrix protein betaIG-H3 is expressed at myotendinous junctions and supports muscle cell adhesion. Cell Tissue Res. 313, 93–105 (2003). 12838408 10.1007/s00441-003-0743-z

[b7] Escribano J., Hernando N., Ghosh S., Crabb J. & Coca-Prados M. cDNA from human ocular ciliary epithelium homologous to beta ig-h3 is preferentially expressed as an extracellular protein in the corneal epithelium. J. Cell. Physiol. 160, 511–521 (1994). 8077289 10.1002/jcp.1041600314

[b8] Skonier J. *et al.* beta ig-h3: a transforming growth factor-beta-responsive gene encoding a secreted protein that inhibits cell attachment *in vitro* and suppresses the growth of CHO cells in nude mice. DNA Cell Biol. 13, 571–584 (1994). 8024701 10.1089/dna.1994.13.571

[b9] Mashima Y. *et al.* Severe form of juvenile corneal stromal dystrophy with homozygous R124H mutation in the keratoepithelin gene in five Japanese patients. Br J Ophthalmol. 82, 1280–1284 (1998). 9924333 10.1136/bjo.82.11.1280 PMC1722402

[b10] Okada M. *et al.* Granular corneal dystrophy with homozygous mutations in the kerato-epithelin gene. Am J Ophthalmol. 126, 169–176 (1998). 9727509 10.1016/s0002-9394(98)00075-0

[b11] Kannabiran C. & Klintworth G. K. *TGFBI* gene mutations in corneal dystrophies. Human Mutation 27 615–625 (2006). 16683255 10.1002/humu.20334

[b12] Aldave A. J. & Sonmez B. Elucidating the molecular genetic basis of the corneal dystrophies: are we there yet? Arch Ophthalmol. 125, 177–186 (2007). 17296893 10.1001/archopht.125.2.177

[b13] Lakshminarayanan R. *et al.* Structure-Function Relationship of TGFβIp (BIGH3) Associated Mutants in Corneal Dystrophy. Brit. J. Ophthalmol. 95(10), 1457–62 (2011). 21835759 10.1136/bjophthalmol-2011-300651

[b14] Ołdak M. *et al.* Late-onset lattice corneal dystrophy without typical lattice lines caused by a novel mutation in the TGFBI gene. Cornea 33(3), 294–9 (2014). 24473223 10.1097/ICO.0000000000000062

[b15] Klintworth G. K., Valnickova Z. & Enghild J. J. Accumulation of beta ig-h3 gene product in corneas with granular dystrophy. Am J Pathol 152, 743–748 (1998). 9502416 PMC1858399

[b16] Klintworth G. K., Bao W. & Afshari N. A. Two mutations in the *TGFBI* (BIGH3) gene associated with lattice corneal dystrophy in an extensively studied family. Invest Ophthalmol Vis Sci. 45, 1382–8 (2004). 15111592 10.1167/iovs.03-1228

[b17] Chiti F., Stefani M., Taddei N., Ramponi G. & Dobson C. M. Rationalization of the effects of mutations on peptide and protein aggregation rates. Nature 424(6950), 805–8 (2003). 12917692 10.1038/nature01891

[b18] Yuan C., Berscheit H. L. & Huang A. J. Identification of an amyloidogenic region on keratoepithelin via synthetic peptides. FEBS Lett 581, 241–247 (2007). 17207483 10.1016/j.febslet.2006.12.019

[b19] Schmitt-Bernard C. F. *et al.* BIGH3 (*TGFBI*) Arg124 mutations influence the amyloid conversion of related peptides *in vitro*. Eur J Biochem 269, 5149–5156 (2002). 12392546 10.1046/j.1432-1033.2002.03205.x

[b20] Clout N. J. & Hohenester E. A model of FAS1 domain 4 of the corneal protein beta(ig)-h3 gives a clearer view on corneal dystrophies. Mol Vis 9, 440–448 (2003). 14502125

[b21] Elavazhagan M. *et al.* Expression, purification and characterization of fourth FAS1 domain of TGFβIp-associated corneal dystrophic mutants. Protein Expr Purif. 84(1), 108–15 (2012). 22575305 10.1016/j.pep.2012.04.018

[b22] Atchaneeyasakul L. O. *et al.* Study Group. A novel H572R mutation in the transforming growth factor-beta-induced gene in a Thai family with lattice corneal dystrophy type I. Jpn J Ophthalmol. 50(5), 403–8 (2006). 17013691 10.1007/s10384-006-0357-6

[b23] Romero P., Moraga M. & Herrera L. Different phenotypes of lattice corneal dystrophy type I in patients with 417C > T (R124C) and 1762A > G (H572R) mutations in TGFBI (BIGH3). Mol Vis. 16, 1601–9 (2010). 20806046 PMC2927433

[b24] Munier F. L. *et al.* BIGH3 mutation spectrum in corneal dystrophies. Invest Ophthalmol Vis Sci. 43, 949–954 (2002). 11923233

[b25] Fujiki K., Nakayasu K. & Kanai A. Corneal dystrophies in Japan. J Hum Genet 46, 431–435 (2001). 11501939 10.1007/s100380170041

[b26] Chakravarthi S. V. V. K., Kannabiran C., Sridhar M. S. & Vemuganti G. K. *TGFBI* gene mutations causing lattice and granular corneal dystrophies in Indian patients. Invest Ophthalmol Vis Sci 46, 121–125 (2005). 15623763 10.1167/iovs.04-0440

[b27] Dighiero P. *et al.* Clinical, histologic, and ultrastructural features of the corneal dystrophy caused by the R124L mutation of the *BIGH3* gene. Ophthalmology 107(7), 1353–7 (2000). 10889112 10.1016/s0161-6420(00)00149-4

[b28] Kocak-Altintas A. G., Kocak-Midillioglu I., Akarsu A. N. & Duman S. *BIGH3* gene analysis in the differential diagnosis of corneal dystrophies. Cornea 20(1), 64–8 (2001). 11189007 10.1097/00003226-200101000-00013

[b29] Wu J. W. *et al.* Comparative analysis of human γD-crystallin aggregation under physiological and low pH conditions. PLoS One. 12, 9(11) (2014). 10.1371/journal.pone.0112309 PMC4229192 25389780

[b30] Noormägi A., Valmsen K., Tõugu V. & Palumaa P. Insulin Fibrillization at Acidic and Physiological pH Values is Controlled by Different Molecular Mechanisms. Protein J. 2015 Oct 22 [Epub ahead of print]. 10.1007/s10930-015-9634-x 26493286

[b31] Kim B. Y. *et al.* Corneal dystrophy-associated R124H mutation disrupts *TGFB*I interaction with Periostin and causes mislocalization to the lysosome. J Biol Chem. 284(29), 19580–91 (2009). 19478074 10.1074/jbc.M109.013607 PMC2740584

[b32] Andreasen M. *et al.* Polymorphic fibrillation of the destabilized fourth fasciclin-1 domain mutant A546T of the Transforming growth factor-β-induced protein (TGFBIp) occurs through multiple pathways with different oligomeric intermediates. J Biol Chem. 287(41), 34730–42 (2012). 22893702 10.1074/jbc.M112.379552 PMC3464576

[b33] Ahmad B., Winkelmann J., Tiribilli B. & Chiti F. Searching for conditions to form stable protein oligomers with amyloid-like characteristics: The unexplored basic pH. Biochim Biophys Acta. 1804(1), 223–34 (2010). 19836473 10.1016/j.bbapap.2009.10.005

[b34] Campioni S. *et al.* Conformational properties of the aggregation precursor state of HypF-N. J Mol Biol. 379(3), 554–67 (2008). 18466920 10.1016/j.jmb.2008.04.002

[b35] Biancalana M. & Koide S. Molecular mechanism of thioflavin-T binding to amyloid fibrils. Biochim. Biophys. Acta. 1804, 1405–1412 (2010). 20399286 10.1016/j.bbapap.2010.04.001 PMC2880406

[b36] Groenning M. Binding mode of thioflavin T and other molecular probes in the context of amyloid fibrils. Current status. J. Chem. Biol. 3, 1–18 (2010). 19693614 10.1007/s12154-009-0027-5 PMC2816742

[b37] Maezawa I. *et al.* Congo red and thioflavin-T analogs detect Abeta oligomers. J Neurochem. 104(2), 457–68 (2008). 17953662 10.1111/j.1471-4159.2007.04972.x

[b38] Grothe H. L. *et al.* Altered protein conformation and lower stability of the dystrophic transforming growth factor beta-induced protein mutants. Mol Vis. 19, 593–603 (2013). 23559853 PMC3611947

[b39] Sasahara K., Yagi H., Naiki H. & Goto Y. Heat-induced Conversion of β2-Microglobulin and Hen Egg-white Lysozyme into Amyloid Fibrils J. Mol. Biol. 372, 981–991 (2007). 17681531 10.1016/j.jmb.2007.06.088

[b40] Cardoso I. *et al.* Transthyretin fibrillogenesis entails the assembly of monomers: a molecular model for *in vitro* assembled transthyretin amyloid-like fibrils. J Mol Biol 317, 683–95 (2002). 11955017 10.1006/jmbi.2002.5441

[b41] Smith D. P., Jones S., Serpell L. C., Sunde M. & Radford S. E. A systematic investigation into the effect of protein destabilisation on beta 2-microglobulin amyloid formation. J Mol Biol 330, 943–54 (2003). 12860118 10.1016/s0022-2836(03)00687-9

[b42] Aso Y., Shiraki K. & Takagi M. Systematic analysis of aggregates from 38 kinds of non disease-related proteins: identifying the intrinsic propensity of polypeptides to form amyloid fibrils. Biosci Biotechnol Biochem 71, 1313–21 (2007). 17485839 10.1271/bbb.60718

[b43] Pfefferkorn C. M., McGlinchey R. P. & Lee J. C. Effects of pH on aggregation kinetics of the repeat domain of a functional amyloid, Pmel17. Proc Natl Acad Sci USA 107(50), 21447–52 (2010). 21106765 10.1073/pnas.1006424107 PMC3003087

[b44] Karring H. *et al.* Differential expression and processing of transforming growth factor beta induced protein (TGFBIp) in the normal human cornea during postnatal development and aging. Exp Eye Res. 90(1), 57–62 (2010). 19788893 10.1016/j.exer.2009.09.011 PMC2789201

[b45] Brunk U. T. & Ericsson J. L. Cytochemical evidence for the leakage of acid phosphatase through utrastructurally intact lysosomal membranes. Histochem J 4, 479–491 (1972). 4120446 10.1007/BF01011128

[b46] Schneider L., Korber A., Grabbe S. & Dissemond J. Influence of pH on wound-healing: a new perspective for wound therapy ? Archives of Dermatological Research 298, 413–420 (2007). 17091276 10.1007/s00403-006-0713-x

[b47] Huynh K. K. & Grinstein S. Regulation of vacuolar pH and its modulation by some microbial species. Microbiol Mol Biol Rev. 71, 452–462 (2007). 17804666 10.1128/MMBR.00003-07 PMC2168644

[b48] Runager K. *et al.* Human phenotypically distinct *TGFBI* corneal dystrophies are linked to the stability of the fourth FAS1 domain of TGFBIp. J. Biol Chem. 286(7), 4951–8 (2011). 21135107 10.1074/jbc.M110.181099 PMC3037607

[b49] Underhaug J. *et al.* Mutation in transforming growth factor beta induced protein associated with granular corneal dystrophy type 1 reduces the proteolytic susceptibility through local structural stabilization. Biochimica et Biophysica Acta 1834, 2812–2822 (2013). 24129074 10.1016/j.bbapap.2013.10.008 PMC4162128

[b50] Klein W. L., Krafft G. A. & Finch C. Targeting small Aβ oligomers: the solution to an Alzheimer’s disease conundrum? Trends Neurosci. 24, 219–224 (2001). 11250006 10.1016/s0166-2236(00)01749-5

[b51] Ferreira S. T., Vieira M. N. & De Felice F. G. Soluble protein oligomers as emerging toxins in Alzheimer’s and other amyloid diseases. IUBMB Life 59, 332–345 (2007). 17505973 10.1080/15216540701283882

[b52] Volles M. J. & Lansbury P. T. Jr. Vesicle permeabilization by protofibrillar alpha-synuclein is sensitive to Parkinson’s disease-linked mutations and occurs by a pore-like mechanism. Biochemistry 41(14), 4595–602 (2002). 11926821 10.1021/bi0121353

[b53] Sokolov Y. *et al.* Soluble amyloid oligomers increase bilayer conductance by altering dielectric structure. J Gen Physiol. 128(6), 637–47 (2006). 17101816 10.1085/jgp.200609533 PMC2151594

[b54] Valincius G. *et al.* Soluble amyloid beta-oligomers affect dielectric membrane properties by bilayer insertion and domain formation: implications for cell toxicity. Biophys J. 95(10), 4845–61 (2008). 18515395 10.1529/biophysj.108.130997 PMC2576380

[b55] Srinivasan S. *et al.* Pathogenic serum amyloid A 1.1 shows a long oligomer-rich fibrillation lag phase contrary to the highly amyloidogenic non-pathogenic SAA2.2. J Biol Chem. 288(4), 2744–55 (2013). 23223242 10.1074/jbc.M112.394155 PMC3554940

[b56] Pace C. N. & Sholtz J. M. Protein Structure: A Practical Approach (Creighton T. E. ed) pp. 299–321, IRL Press, Oxford, UK.

[b57] Benjwal S., Verma S., Rohm K. H. & Gursky O. Monitoring protein aggregation during thermal unfolding in circular dichroism experiments. Protein science: a publication of the Protein Society 15, 635–9 (2006). 16452626 10.1110/ps.051917406 PMC2249783

[b58] Bullock A. N. *et al.* Thermodynamic stability of wild-type and mutant p53 core domain. Proc Natl Acad Sci USA 94, 14338–42 (1997). 9405613 10.1073/pnas.94.26.14338 PMC24967

[b59] Pace C. N. & Shaw K. L. Linear extrapolation method of analyzing solvent denaturation curves. Proteins Suppl 4, 1–7 (2000). 11013396 10.1002/1097-0134(2000)41:4+<1::aid-prot10>3.3.co;2-u

[b60] Urcan E. *et al.* Real-time xCELLigence impedance analysis of the cytotoxicity of dental composite components on human gingival fibroblasts. Dental Materials 26, 51–58 (2010). 19767088 10.1016/j.dental.2009.08.007

