# Supplementary material for: pH Induced Conformational Transitions in the Transforming Growth Factor β-Induced Protein (TGFβIp) Associated Corneal Dystrophy Mutants
Source: Sci Rep. 2016 Mar 31;6:23836. doi: 10.1038/srep23836 (PMC4814907; doi:10.1038/srep23836)
Supplement: Supplementary Figures [file srep23836-s1.pdf]

pH Induced Conformational Transitions in the Transforming Growth Factor β-Induced Protein (TGFβIp) Associated Corneal Dystrophy Mutants

Elavazhagan Murugan, Anandalakshmi Venkatraman, Zhou Lei, Victoria Mouvet, Rayne Rui Yi Lim, Nandhakumar Muruganantham, Eunice Goh, Gary Swee Lim Peh, Roger W. Beuerman, Shyam S. Chaurasia, Lakshminarayanan Rajamani*, Jodhbir S. Mehta*

* To whom correspondence should be addressed

Supplementary Figures

Supplementary figures

Figure legend

Fig S1. Effects of pH on the aggregation and oligomerization of the 4th FAS1 domains of TGFβIp. (a-c). ThT fluorescence, The 4th FAS1 domains of TGFβIp WT and mutants were incubated in acidic pH conditions (along with 30μM ThT) for 1 week and their possible oligomerization or conversion to β-sheet oligomers was examined by measuring their emission fluorescence at 485 nm. (d-f). CD. The corresponding CD spectra for the domains before and after incubation. Compared to the amyloid fibril peptide pN622K, almost little or no fluorescence was observed in WT and R555W. The slight increase in fluorescence corresponding to pH 2.75 and pH 3.25 for H572R did not show a corresponding conversion in the CD spectra.

Fig S2. Thermal Denaturation from 20 °C to 90 °C. Variable temperature CD curves at 222 nm of WT (black), R555W (red) and H572R (blue) proteins heated from 20 °C to 90 °C at various pH (3.0 [a], 4.5 [b], 5.5 [c], 7.0 [d] and 8.0 [e]) and the CD intensities at 222 nm were plotted as a function of temperature. The baseline subtracted curves of the WT (black), R555W (red) and H572R (blue) proteins show that while there was no transition observed in the WT in all the conditions as observed from the unchanged straight line in black, little or no changes were seen in pH 7 and pH 8 for the mutants. However, clear transitions to β-sheet were observed at acidic pH (pH 3, pH 4.5 and pH 5.5) for both the mutants.

Figure S3 Urea Denaturation and Renaturation. (a-d) Fluorescence emission spectra of R555W showing the reversibility to folded state after removal of urea. The R555W mutant was incubated with 8M Urea for 24 hours at various acidic conditions (pH 3, pH 4.5, pH 5.5) and pH 7 and the emission fluorescence before and after urea incubation was measured. The emission spectra before urea incubation 332 nm (black), after incubating with 8M urea (red) and after removing urea by buffer exchange (blue). Unfolding of the protein is seen by the shifting of peaks from ~332 nm to ~ 352 nm. The refolding of the protein after removal of urea is seen by the return of the emission maximum to ~332 nm. The decrease in the emission intensities result from change in volumes and corresponding decrease in concentration following buffer exchange. (e-h) Change in the fluorescence emission maxima from ~332nm to ~352nm of R555W with increase in urea concentration (shown as black squares) and the reversibility of the fluorescence emission maxima from ~352nm to ~332nm after gradual removal of urea (shown as red circles).

Fig S4. Seeded Fibrillation studies. The 4th FAS1 domains of TGFβIp WT and mutants were incubated in acidic pH conditions (pH 2.0, pH 2.75, pH 3.25, pH 4.0, pH 4.5 and pH 5.25) and pH 7.0 along with preformed β-oligomers from the respective domains (β-oligomers of H572R for WT). After 2 weeks, the samples were incubated with 30μM ThT and their fluorescence measured. While, the amyloid peptide fibril pN622K displayed high fluorescence intensity, no significant fluorescence was observed for the other samples.

Figure S1

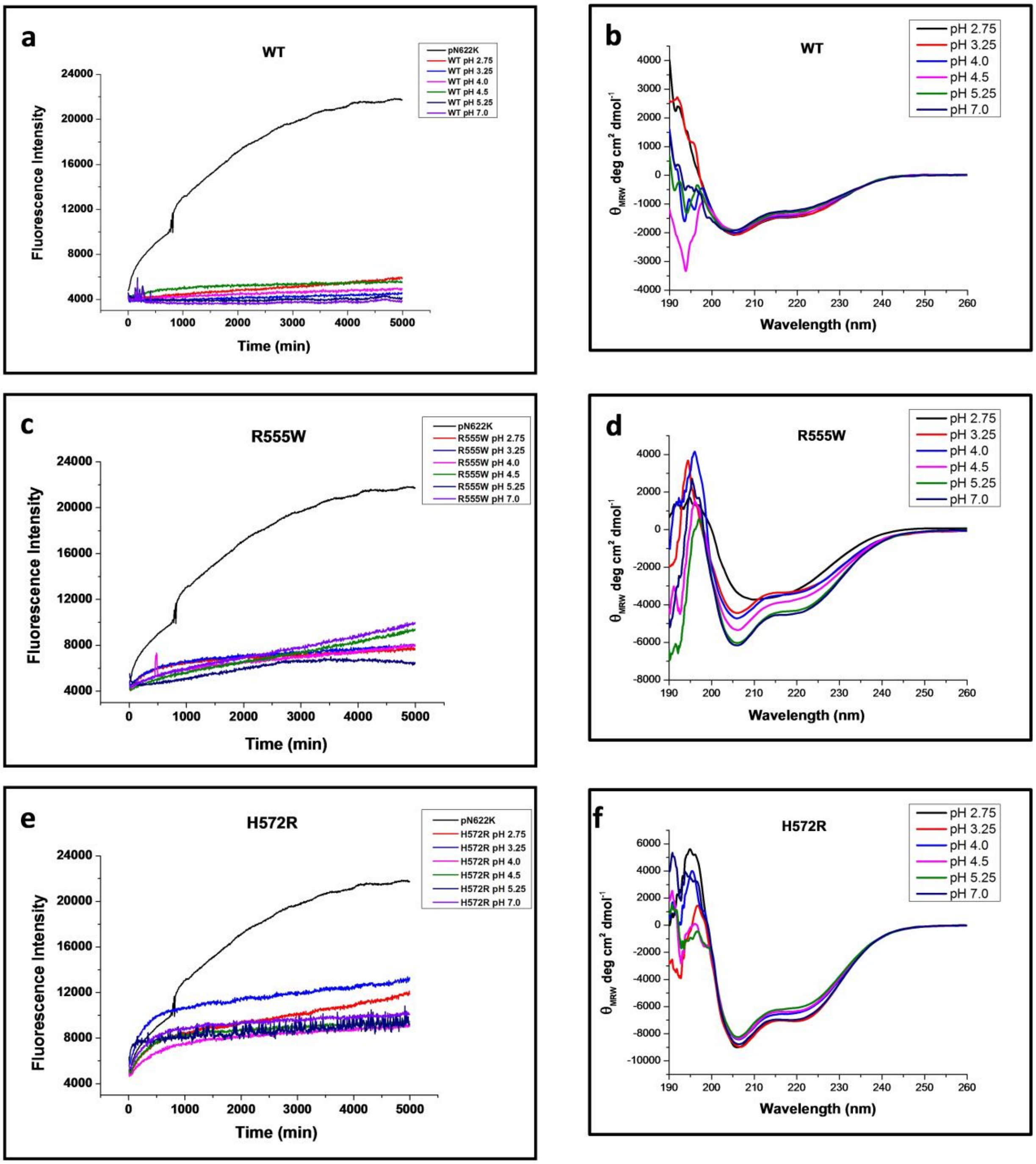
a
WT
pN622K
WT pH 2.75
WT pH 3.25
WT pH 4.0
WT pH 4.5
WT pH 5.25
WT pH 7.0
Fluorescence Intensity
Time (min)
b
WT
pH 2.75
pH 3.25
pH 4.0
pH 4.5
pH 5.25
pH 7.0
θ_MRW deg cm² dmol⁻¹
Wavelength (nm)
c
R555W
pN622K
R555W pH 2.75
R555W pH 3.25
R555W pH 4.0
R555W pH 4.5
R555W pH 5.25
R555W pH 7.0
Fluorescence Intensity
Time (min)
d
R555W
θ_MRW deg cm² dmol⁻¹
Wavelength (nm)
e
H572R
pN622K
H572R pH 2.75
H572R pH 3.25
H572R pH 4.0
H572R pH 4.5
H572R pH 5.25
H572R pH 7.0
Fluorescence Intensity
Time (min)
f
H572R
θ_MRW deg cm² dmol⁻¹
Wavelength (nm)

Figure S2

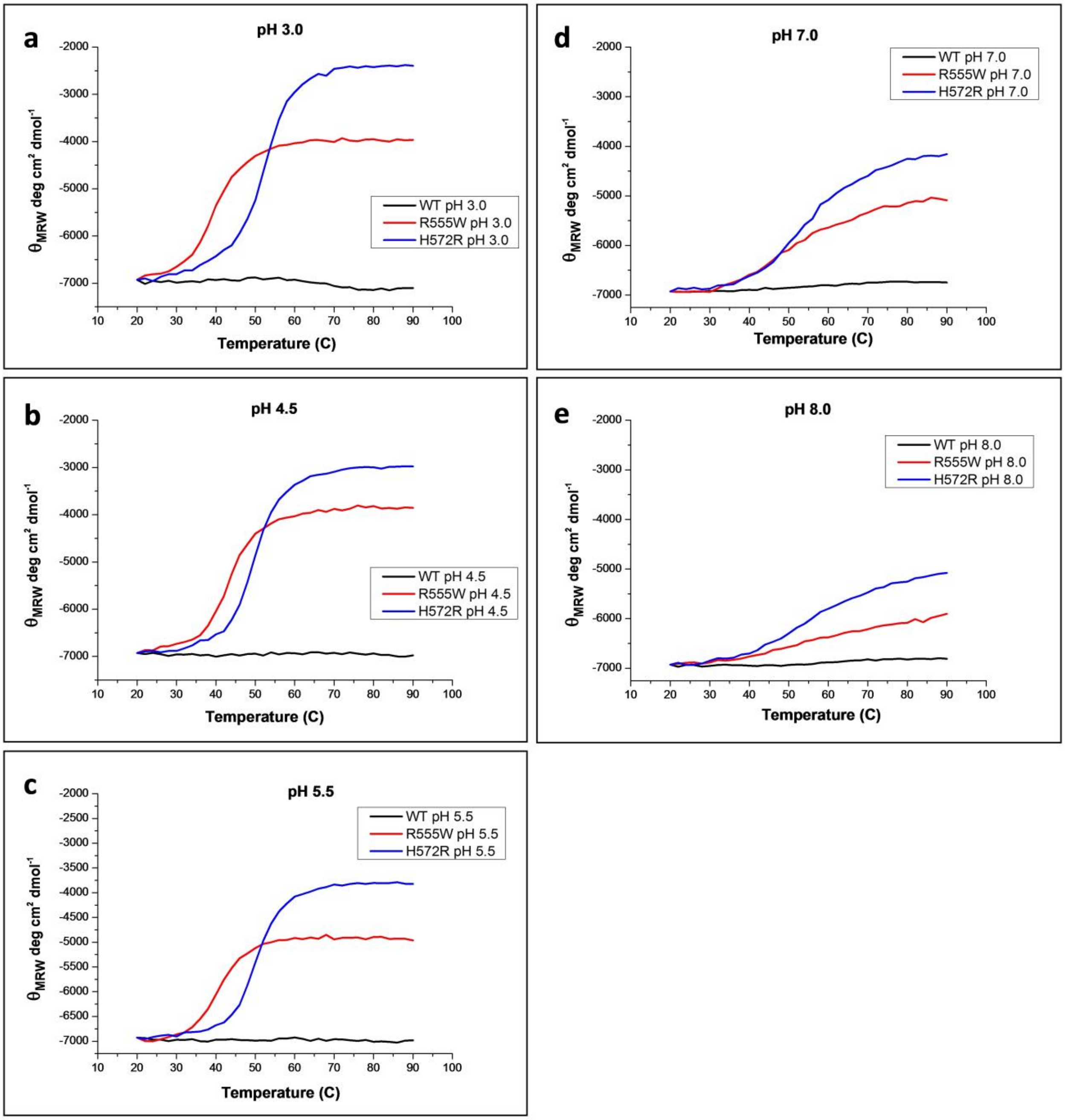

a
pH 3.0
WT pH 3.0
R555W pH 3.0
H572R pH 3.0
b
pH 4.5
WT pH 4.5
R555W pH 4.5
H572R pH 4.5
c
pH 5.5
WT pH 5.5
R555W pH 5.5
H572R pH 5.5
d
pH 7.0
WT pH 7.0
R555W pH 7.0
H572R pH 7.0
e
pH 8.0
WT pH 8.0
R555W pH 8.0
H572R pH 8.0
θ_{MRW} deg cm² dmol⁻¹
Temperature (C)

Figure S3

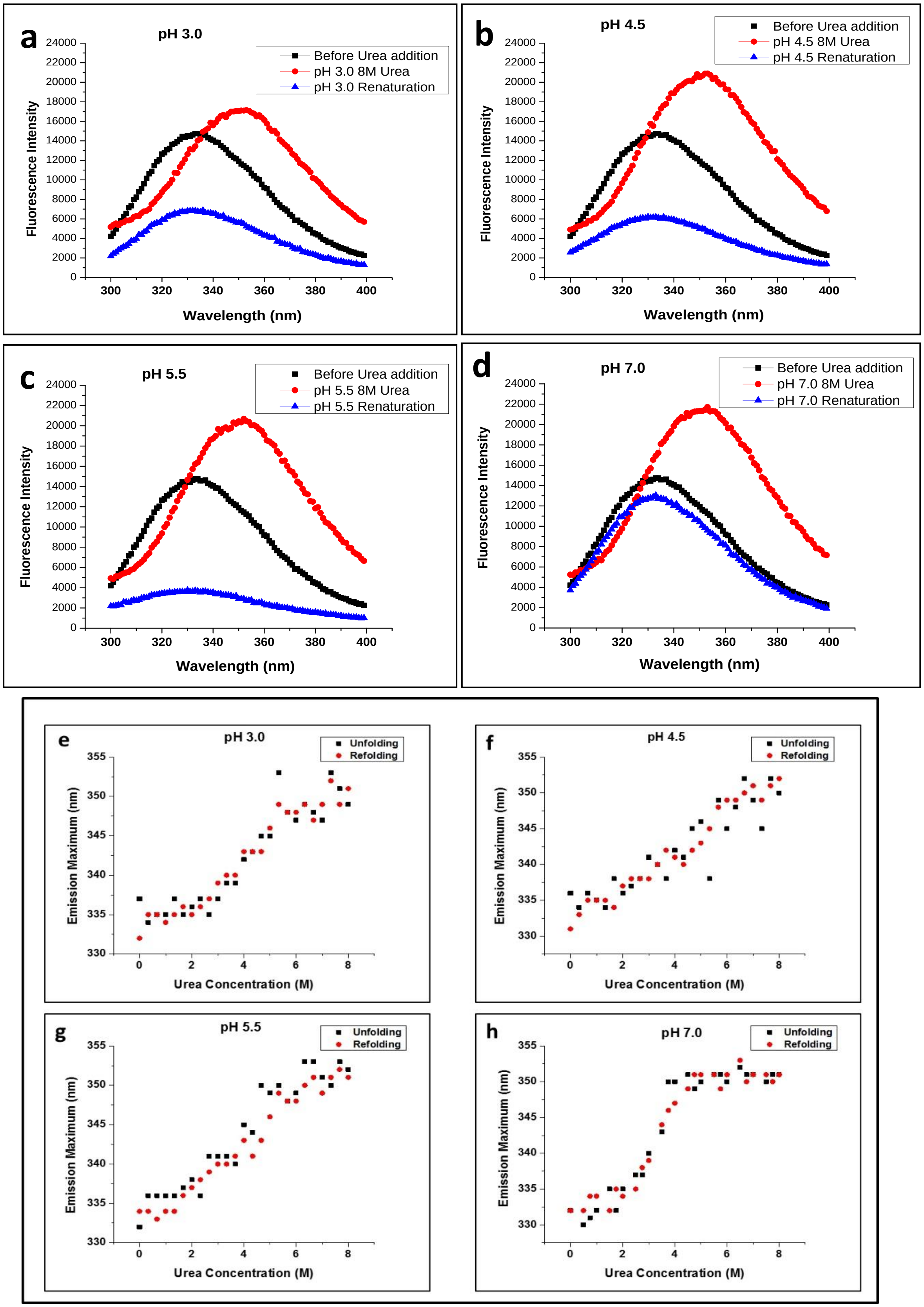

a
pH 3.0
Before Urea addition
pH 3.0 8M Urea
pH 3.0 Renaturation
b
pH 4.5
Before Urea addition
pH 4.5 8M Urea
pH 4.5 Renaturation
c
pH 5.5
Before Urea addition
pH 5.5 8M Urea
pH 5.5 Renaturation
d
pH 7.0
Before Urea addition
pH 7.0 8M Urea
pH 7.0 Renaturation
Fluorescence Intensity
Wavelength (nm)
e
pH 3.0
f
pH 4.5
g
pH 5.5
h
pH 7.0
Unfolding
Refolding
Emission Maximum (nm)
Urea Concentration (M)

Figure S4

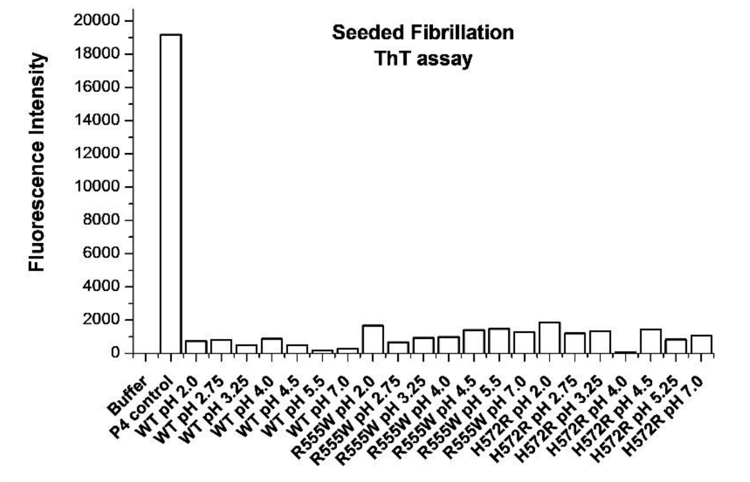

Seeded Fibrillation
ThT assay
Fluorescence Intensity
20000
18000
16000
14000
12000
10000
8000
6000
4000
2000
0
Buffer
P4 control
WT pH 2.0
WT pH 2.75
WT pH 3.25
WT pH 4.0
WT pH 4.5
WT pH 5.5
WT pH 7.0
R555W pH 2.0
R555W pH 2.75
R555W pH 3.25
R555W pH 4.0
R555W pH 4.5
R555W pH 5.5
R555W pH 7.0
H572R pH 2.0
H572R pH 2.75
H572R pH 3.25
H572R pH 4.0
H572R pH 4.5
H572R pH 5.25
H572R pH 7.0
