# Supplementary material for: pH Induced Conformational Transitions in the Transforming Growth Factor β-Induced Protein (TGFβIp) Associated Corneal Dystrophy Mutants
Source: Sci Rep. 2016 Mar 31;6:23836. doi: 10.1038/srep23836 (PMC4814907; doi:10.1038/srep23836)

**pH Induced Conformational Transitions in the Transforming Growth Factor β-Induced Protein (TGFβIp) Associated Corneal Dystrophy Mutants**

Elavazhagan Murugan, Anandalakshmi Venkatraman, Zhou Lei, Victoria Mouvet, Rayne Rui Yi Lim, Nandhakumar Muruganantham, Eunice Goh, Gary Swee Lim Peh, Roger W. Beuerman, Shyam S. Chaurasia, Lakshminarayanan Rajamani^*^, Jodhbir S. Mehta^*^

* To whom correspondence should be addressed

**Supplementary Data**

LC-MS Spectra for the 4^th^ FAS1 domains of TGFβIp WT, and the native proteins and β-oligomers of R555W and H572R mutants.


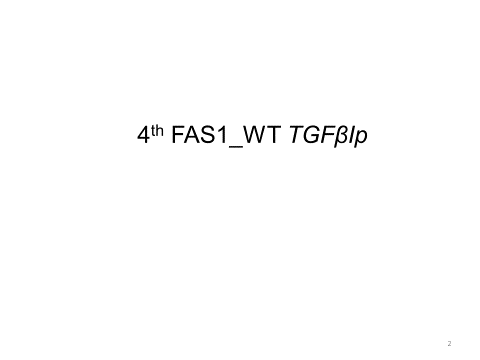


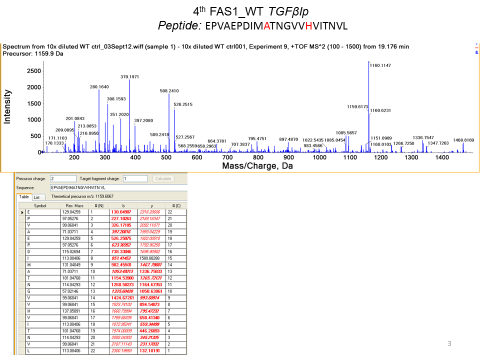


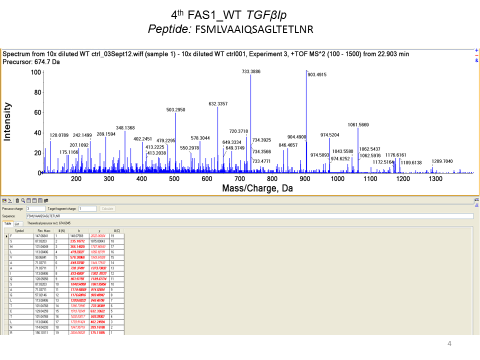


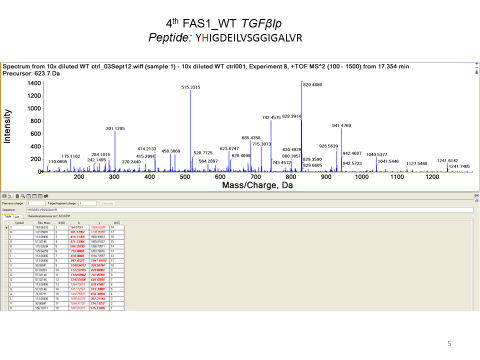

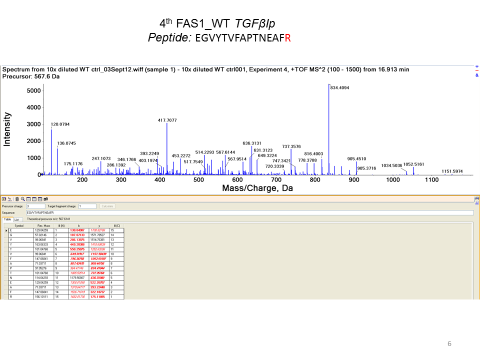

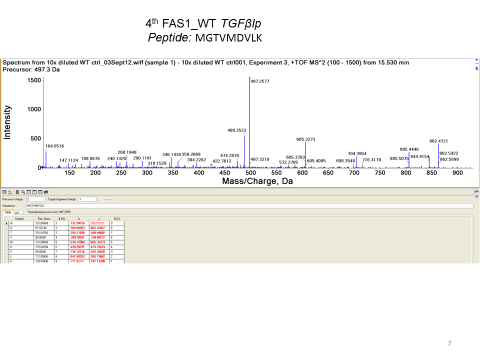

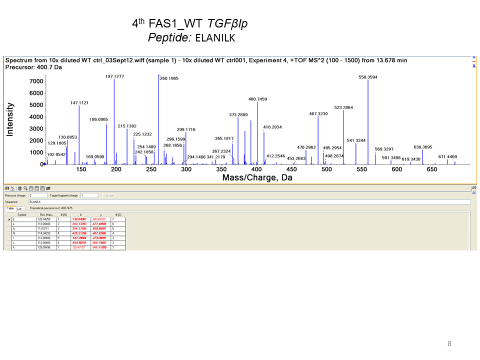

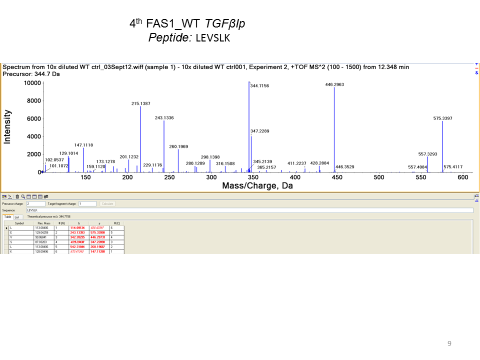

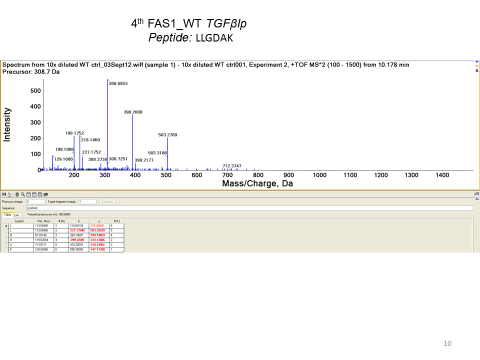

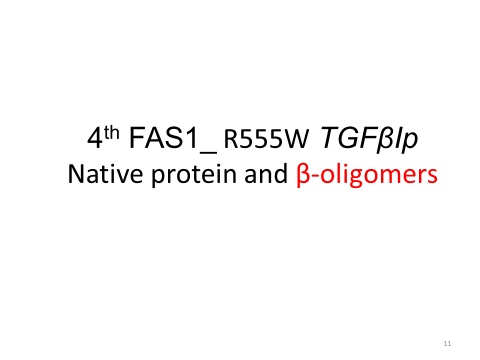


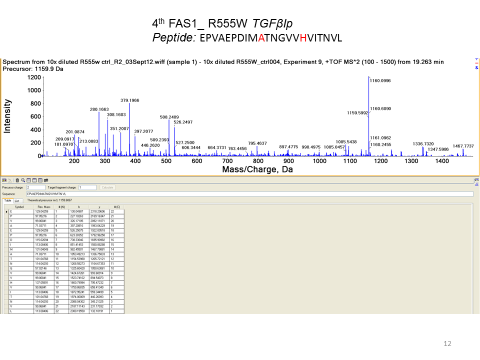

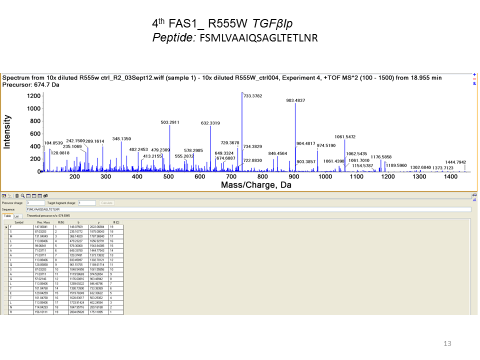

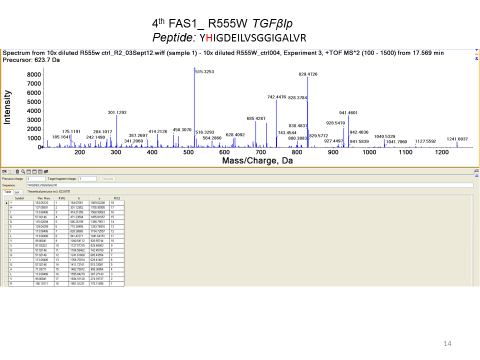

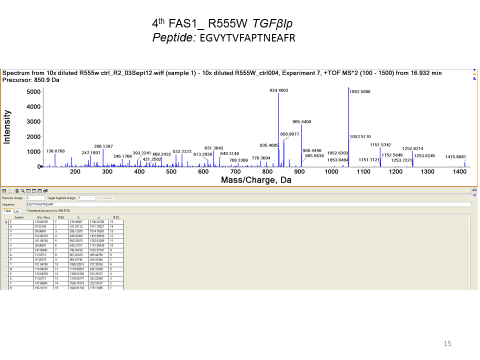

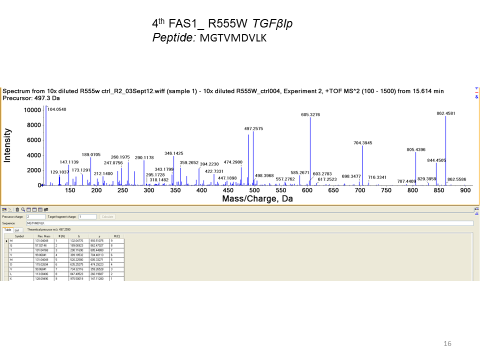

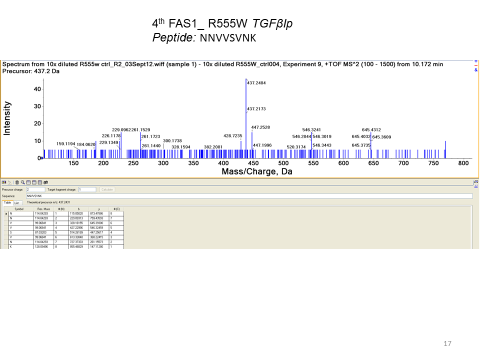

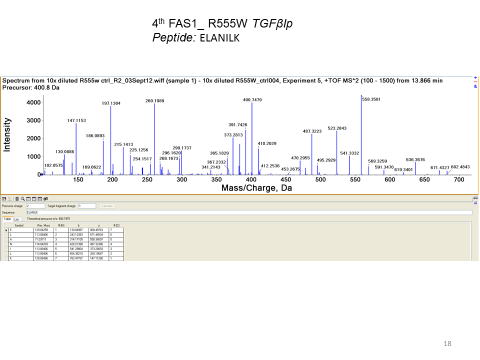

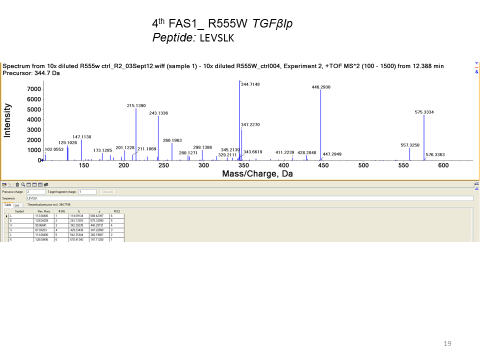

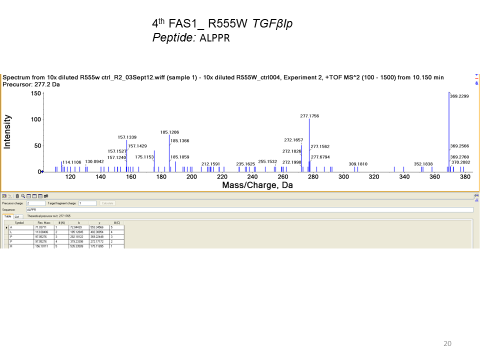

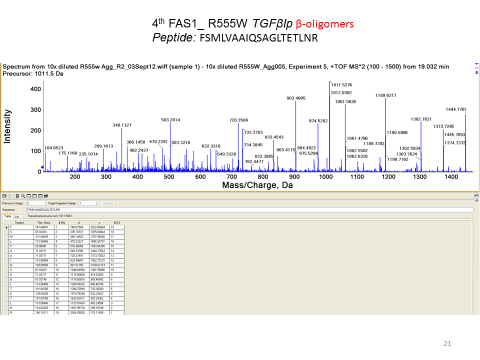

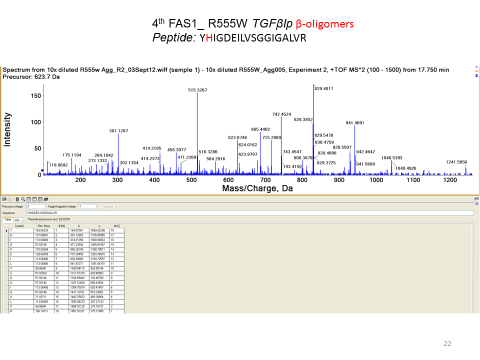

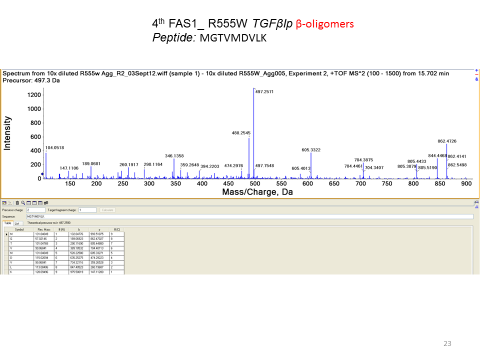

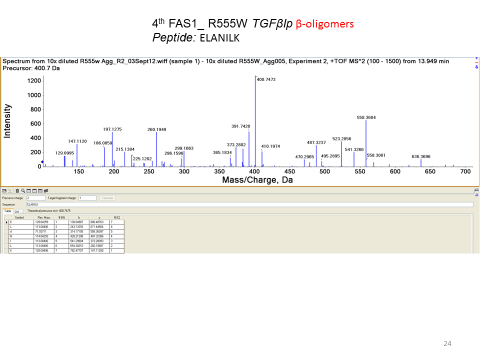

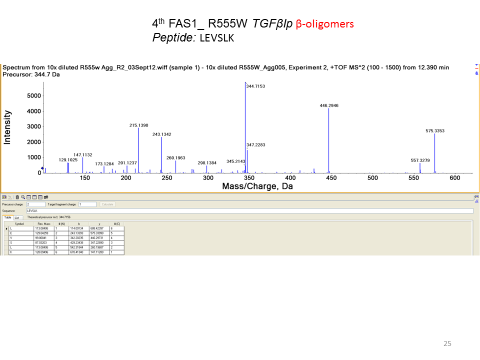

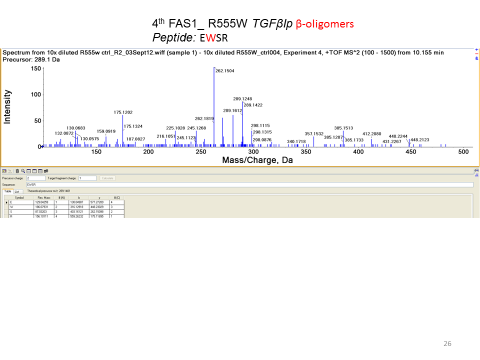


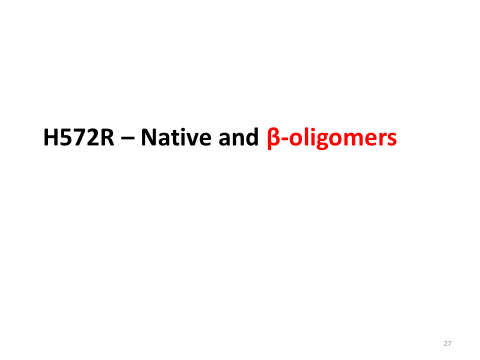


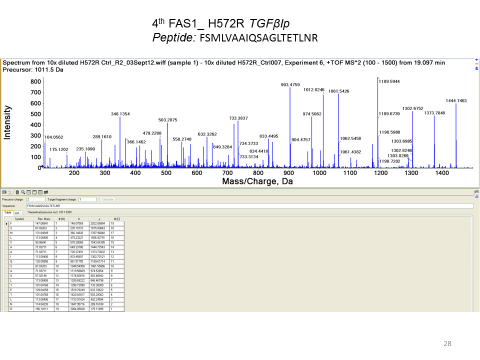

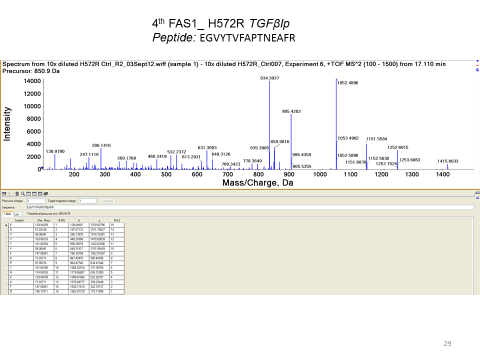

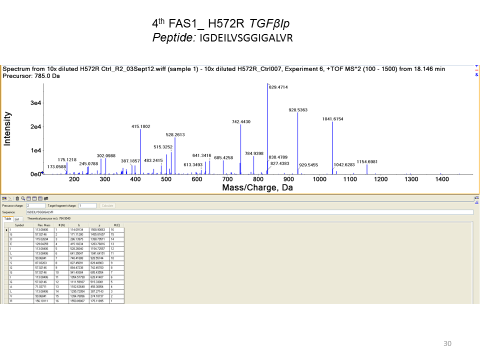

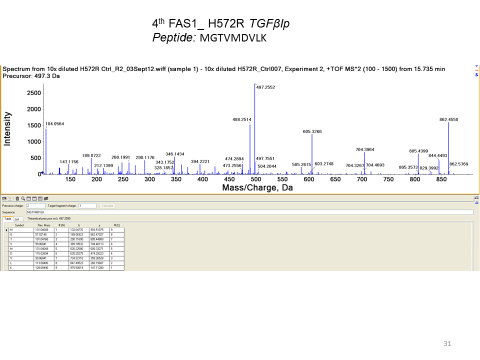

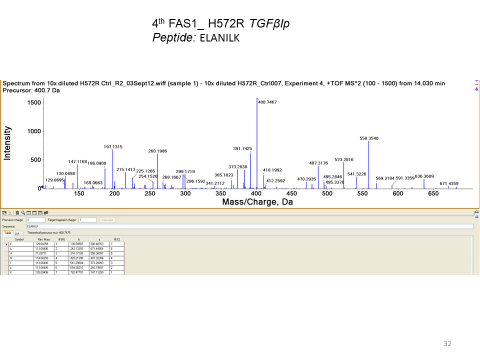

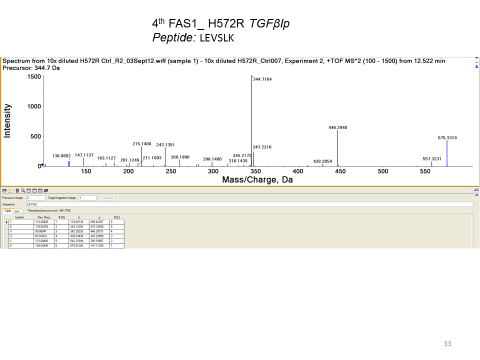

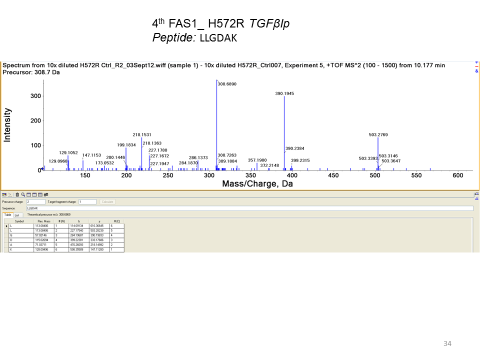

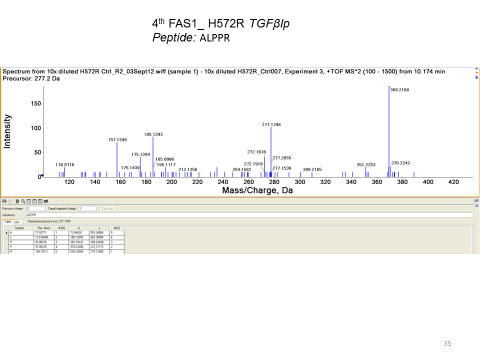

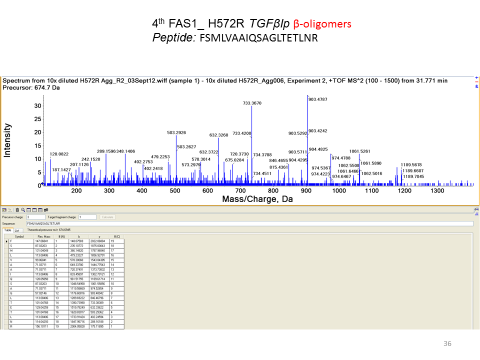

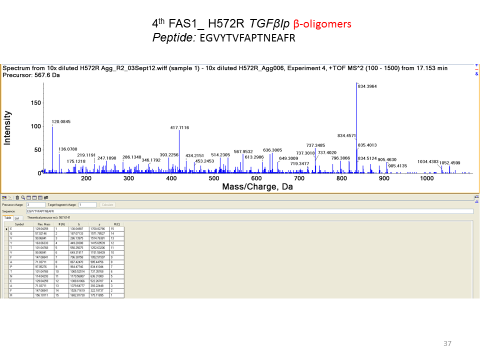

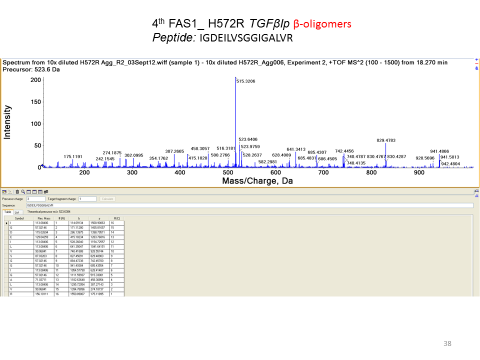

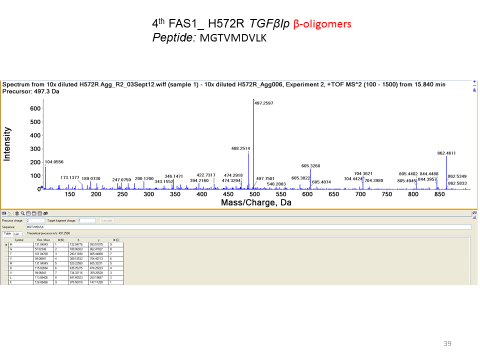

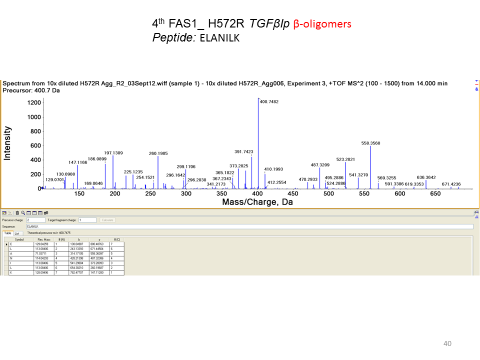

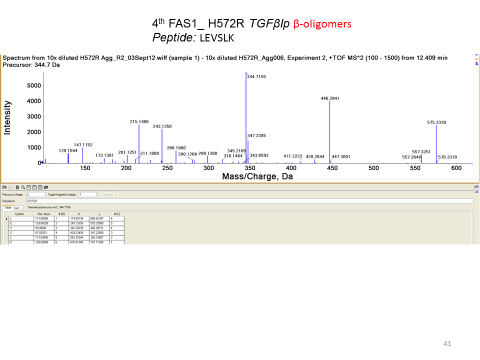

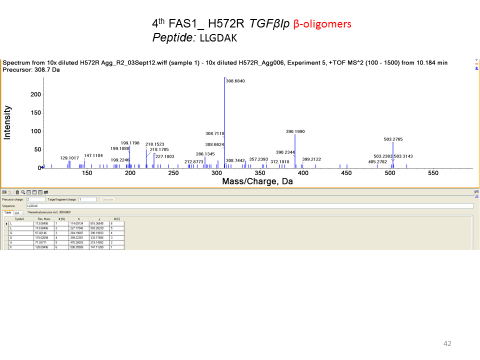

Supplement: Supplementary Data [file srep23836-s2.doc]
